# Aptamer-guided gene targeting in yeast and human cells

**DOI:** 10.1093/nar/gku101

**Published:** 2014-02-05

**Authors:** Patrick Ruff, Kyung Duk Koh, Havva Keskin, Rekha B. Pai, Francesca Storici

**Affiliations:** School of Biology, Georgia Institute of Technology, Atlanta, GA, 30332, USA

## Abstract

Gene targeting is a genetic technique to modify an endogenous DNA sequence in its genomic location via homologous recombination (HR) and is useful both for functional analysis and gene therapy applications. HR is inefficient in most organisms and cell types, including mammalian cells, often limiting the effectiveness of gene targeting. Therefore, increasing HR efficiency remains a major challenge to DNA editing. Here, we present a new concept for gene correction based on the development of DNA aptamers capable of binding to a site-specific DNA binding protein to facilitate the exchange of homologous genetic information between a donor molecule and the desired target locus (aptamer-guided gene targeting). We selected DNA aptamers to the I-SceI endonuclease. Bifunctional oligonucleotides containing an I-SceI aptamer sequence were designed as part of a longer single-stranded DNA molecule that contained a region with homology to repair an I-SceI generated double-strand break and correct a disrupted gene. The I-SceI aptamer-containing oligonucleotides stimulated gene targeting up to 32-fold in yeast *Saccharomyces cerevisiae* and up to 16-fold in human cells. This work provides a novel concept and research direction to increase gene targeting efficiency and lays the groundwork for future studies using aptamers for gene targeting.

## INTRODUCTION

Targeted gene modification is a powerful tool for researchers interested in functional analysis of genes and potentially for gene therapy applications. The primary limitation of gene targeting is the low frequency with which it occurs in many organisms and cell types, including mammalian cells, occurring in roughly one cell for every 10^5^–10^7^ treated cells (1). The low frequency of gene targeting, which relies on HR is due in part to the much higher frequency of random integration via non-homologous end joining (NHEJ), which occurs in about 1 cell for every 10^2^–10^4^ treated cells (1).

Several strategies have been used to increase the frequency of gene targeting. It was shown that a DNA double-strand break (DSB) at the target site increases the frequency of gene targeting several orders of magnitude in bacteria (2), yeast (3), plants (4), fruit flies (5), mice (6), human embryonic stem cells (7) and many other cell types. Another strategy to increase gene targeting in mammalian cells has been achieved through the overexpression of key recombination proteins from HR proficient organisms. Overexpression of bacterial RecA led to a 10-fold increase in gene targeting in mouse cells (8); likewise, overexpression of yeast Rad52 led to a 37-fold increase in gene targeting in human cells (9). Conversely, another approach for increasing gene targeting in human cells involves decreasing the amount of DSB repair through the pathway of NHEJ. In mouse embryonic stem cells, an increase in gene targeting was seen in Ku70 (6-fold), XRCC4 (2-fold) and DNAPK-cs-deficient cell lines (2-fold) (10), and a 3-fold increase in Chinese hamster ovary cells lacking DNAPK-cs (11). Similarly, knockdown of Ku70 and XRCC4 in human colon cancer cells led to a 30-fold increase in gene targeting (12).

Different from the methodologies mentioned above that focused on increasing HR or decreasing NHEJ, it was shown that knockout of the *RAD51* recombinase prevents DSB-induced sister chromatid exchange (13), and thus facilitates gene targeting by single-stranded oligonucleotides at the site of a DSB in both haploid and diploid yeast systems (14). Gene correction close to a DSB by single-stranded oligonucleotides does not require Rad51, but only the strand annealing function of Rad52 (14). Thus, deleting Rad51 favours DSB-driven recombination by oligonucleotides by strongly reducing the competition with the sister chromatid and/or the homologous chromosome for DSB repair (13–15). Similarly, it was shown that by knocking down human SMC1, important for HR between sister chromatids, gene targeting increases (16). Without proximity to the DSB site, the sister chromatid was used less frequently as a donor, shifting repair of the DSB more towards HR with the exogenous donor sequence.

Here we have developed a novel gene targeting approach, in which we bind a site-specific DNA binding protein by a DNA aptamer to target a donor molecule to a specific genetic locus for correction (aptamer-guided gene targeting [AGT]). DNA aptamers are sequences of DNA that are able to bind to a specific target with high affinity because of their unique secondary structure (17). The AGT approach increases the efficiency of gene targeting by guiding the exogenous donor DNA into the vicinity of the site targeted for genetic modification. By tethering the exogenous donor DNA to the site-specific homing endonuclease I-SceI, which recognizes an 18-bp sequence and generates a DSB (18), we achieved targeted delivery of exogenous donor DNA to the site of the I-SceI DSB in different genomic locations in yeast and human cells, facilitating gene correction by the donor DNA. DNA aptamers were selected for binding to the I-SceI protein using a variant of capillary electrophoresis systematic evolution of ligands by exponential enrichment (CE-SELEX) called ‘Non-SELEX’ (19). By synthesizing a DNA oligonucleotide that contained the I-SceI aptamer sequence as well as homology to repair the I-SceI DSB and correct a target gene, we were able to increase gene targeting frequencies up to 32-fold over a non-binding control in yeast, and up to 16-fold over a non-binding control in human cells. Our strategy offers a novel way to increase gene targeting and represents the first study to use aptamers in the context of gene correction.

## MATERIALS AND METHODS

### Aptamer selection

The protein of interest, I-SceI, was provided by F. Gimble (Purdue University, West Lafayette, IN) in storage buffer (10 mM KPO_4_, pH 7.4, 100 nM EDTA, 1 mM DTT, 100 mM NaCl and 50% glycerol). Before selection, to remove storage buffer components, I-SceI was dialyzed in run buffer 1 (RB1), 50 mM Tris-HCl at pH 8.2, yielding a concentration of 3 µM I-SceI in RB1. RB1 was the run buffer used for the capillary electrophoresis. The DNA library was purchased from Alpha DNA (Montreal, Quebec, Canada) and contained the following sequence: 5′_CTTCTGCCCGCCTCCTTCC-(N)36-GACGAGATAGGCGGACACT_3′ (Supplementary Table S1). The library was composed of a sequence with 36 random nucleotides flanked by two fixed 19-base regions used later as primer sequences for polymerase chain reaction (PCR) amplification using the forward aptamer-amplifying primer P1 (5′_CTTCTGCCCGCCTC CTTCC_3′) and the reverse primer P2 (5′_AGTGTCCGCCTATCTCGTC_3′) (Supplementary Table S1).

The protocol for SELEX using capillary electrophoresis (CE) was essentially as described earlier (20), but with a few modifications. Initial calibrations were done with a serial dilution of the aptamer library in RB1. The free DNA run time with 100 nM of the library was determined (Supplementary Figure S1A). The initial bulk affinity assay was performed with 1.5 µM I-SceI and 100 nM DNA to visualize peaks corresponding to I-SceI-DNA complexes and determine the aptamer collection window from the beginning of the first complex peak to the end of the last complex peak (Supplementary Figure S1B). CE was done using a Beckman Coulter (Atlanta, GA, USA) P/ACE MDQ with laser-induced fluorescence (LIF) detection. The LIF was composed of a 488-nm air-cooled argon ion laser along with an on-board detector. CE runs were carried out with a voltage of 10 kV. The first round of selection began after determination of the collection window based on the bulk affinity analysis. For the initial round of *in vitro* selection, the DNA library (5 µl at 200 µM) was mixed with 5 µl of selection buffer 3 (SB3) (100 mM Tris-HCl at pH 8.2, 200 mM NaCl and 10 mM MgCl_2_) for a final concentration of 100 µM DNA library, 50 mM Tris-HCl at pH 8.2, 100 mM NaCl and 5 mM MgCl_2_. This mixture was heated in the BioRad iCycler to 94°C for 1 min, and then cooled to 20°C at a rate of 0.5°C/s. After the folding of the DNA library, 5 µl of 200 nM I-SceI dissolved in selection buffer 1 (SB1) (50 mM Tris-HCl at pH 8.2, 100 mM NaCl and 5 mM MgCl_2_) was added to 5 µl of the DNA-SB3 mixture to make the final volume to 10 µl. This brought the final concentrations to 50 µM DNA library, 100 nM I-SceI, 100 mM NaCl, 5 mM MgCl_2_ and 50 mM Tris-HCl (pH 8.2). The collection window was from the beginning of the first complex peak to the end of the last complex peak, well before the free DNA peak. The fraction collected was typically 0.3–0.5 µl that was collected into a tube containing 10 µl of the above mixture except without any additional DNA. After 15 min of incubation at room temperature, this new mixture was used in subsequent rounds of selection. Despite the reduction in I-SceI concentration compared with the bulk affinity assay, complexes were still observed for the first round of selection (Supplementary Figure S2A). In the second round of selection, the ratio of DNA forming a complex compared with free DNA was much higher than in the first round (Supplementary Figure S2B). Selection proceeded to a third round; however, no complexes were observed due to the low amount of total DNA (Supplementary Figure S2C). The fraction collected from the second round of CE containing DNA forming a complex with I-SceI was used for subsequent analysis as an aptamer pool.

### Quantitative real-time-PCR and amplification of the aptamer pool

After the aptamer selection, the collected fraction containing the aptamer pool was analysed through quantitative real-time PCR (qRT-PCR) using the ABI (Carlsbad, CA, USA) StepOnePlus Real-Time PCR system. qRT-PCR analysis was essential to determine the optimum number of cycles for subsequent PCR amplification of the aptamer pool. qRT-PCR was done with the forward aptamer-amplifying primer P1 (5′_CTTCTGCCCGCCTCCTTCC_3′) and the reverse primer P2 (5′_AGTGTCCGCCTATCTCGTC_3′), respectively (Supplementary Table S1). The primers were designed using OligoAnalyzer (http://www.idtdna.com/analyzer/Applications/OligoAnalyzer/) to limit complementarity to each other, thus to reduce occurrence of primer dimers during PCR amplification reactions, and were ordered from Eurofins MWG Operon (Huntsville, AL, USA). For amplification, 20 µl of PCR mix was prepared consisting of 10 µl of 2X Quanta SYBR Green PCR Master Mix (Roche, Basel, Switzerland), 0.6 µl of 10 µM P1, 0.6 µl of 10 µM P2, 1 µl of collected fraction as template and 7.8 µl H_2_O. The qRT-PCR set-up included one cycle with a denaturation step at 94°C for 30 s, followed by 50 cycles with a denaturation step at 94°C for 10 s, an annealing step at 55°C for 10 s and an extension step at 72°C for 10 s, followed by another extension at 72°C for 1 min, ending by holding at 4°C.

Following qRT-PCR, the fraction containing the potential aptamers was amplified using standard PCR. PCR was done in a 100 µl volume consisting of 1 µl of 5 U/µl Ex Taq polymerase, 3 µl of 10 µM forward primer P1, 3 µl of 10 µM reverse primer P2, 10 µl of 10× Mg^2+^ buffer (Takara Ex Taq, Clontech Laboratories, Mountainview, CA, USA), 8 µl of 2.5 mM each dNTP, 70 µl H_2_O and 5 µl of the collected fraction from capillary electrophoresis. In a previous protocol to select DNA aptamers, it was shown that over-amplification of the random oligonucleotide library leads to formation of non-specific products (21); therefore, the random library was only amplified to ∼50% of the maximum yield as measured by qRT-PCR.

### Cloning and sequencing

Post-selection DNA cloning of the aptamer pool was done with the TOPO Zero Blunt Cloning Kit (Invitrogen, Grand Island, NY, USA). Standard PCR with unlabelled primers P1 and P2 was used to generate double-stranded DNA containing the aptamer sequence, which was then blunt-end ligated into the PCR-Blunt II-TOPO vector that contains the kanamycin resistance gene. After transformation into *Escherichia coli* DH5α cells, colonies were selected for growth on kanamycin-containing media (kanamycin final concentration was 40 µg/ml). Plasmid DNA was crudely extracted by placing selected colonies into 50 µl of RNase/DNase-free water and incubating in a boiling water bath for 5 min. Debris was pelleted by centrifugation for 10 min at 10 000*g* and the supernatant was used for an asymmetric PCR. Asymmetric PCR, in which the concentration of the P1 primer was 10-fold higher (10 µM) than that of the P2 primer (1 µM), with FAM-labelled primer P1 (P1-FAM) and unlabelled primer P2 were used on the plasmid DNA to predominately generate the strand of interest, which was then analysed using CE with LIF. PCR products were used with 1.5 µM I-SceI in the same manner described previously for CE analysis. Individual plasmids that showed strong binding through their asymmetric PCR products were isolated using the GeneJET Plasmid Miniprep Kit (Thermo Scientific, Pittsburgh, PA, USA) and sequenced by Eurofins MWG Operon (Huntsville, AL, USA). Based on sequencing results, several candidate aptamers were chosen and ordered as salt-free oligonucleotides. Consensus sequence was analysed using ClustalW2 (http://www.ebi.ac.uk/Tools/msa/clustalw2/).

### Electrophoretic mobility shift assay

Potential aptamer oligonucleotides and a negative control oligonucleotide were 5′ labelled with P^32^ γ-ATP using T4 Polynucleotide Kinase (New England Biolabs, Ipswich, MA, USA). The negative control (P1-r-P2) consisted of an oligonucleotide of the same length as the random DNA library (74 bases), contained the same flanking primer regions and had a fixed sequence for its internal region 5′-CTTCTGCCCGCCTCCTTCCGGTCGGGCACACCTGTCATACCCAATCTCGAG GCCAGACGAGATAGGCGGACACT-3′ (Supplementary Table S1). The internal region was chosen using a random DNA sequence generator with a specified GC content of 50% (http://www.faculty.ucr.edu/∼mmaduro/random.htm). Electrophoretic mobility shift assay (EMSA) conditions were as described previously (22), with some modifications. For a more detailed description see Supplementary Materials and Methods.

### Yeast strains

Three different strain backgrounds were used for these studies, BY4742 (*MAT*α *his3*Δ1 *leu2*Δ0 *lys2*Δ0 *ura3*Δ0) (3), 55R5-3C [*MAT*a *ura1 omega*^-^ C321 (chloramphenicol resistant)] (23) and FRO-767 (*leu2*::*HOcs*^1^, *mat***a**Δ::*hisG*, *ho*Δ, *hml*Δ::*ADE1*, *hmr*Δ::*ADE1*, *ade1*, *leu2-3*,*112*, *lys5*, *trp1*::*hisG*, *ura3-52*, *ade3*::*GAL*::*HO*) (24) (Supplementary Table S2). The *TRP5*, *ADE2* and *LEU2* loci were tested in the BY4742 background. The *TRP1*, *ADE2* and *LEU2* loci were tested in the 55R5-3C background. The *TRP5* locus was also tested in the FRO-767 background.

**Figure 1.**
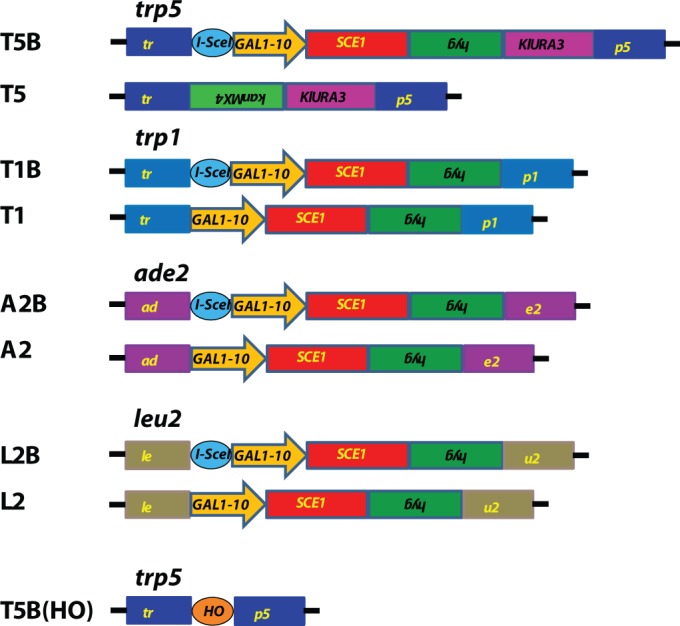
Scheme of targeted yeast loci. The FRO-155/156 strain, shown above as T5B, contains the I-SceI break site (blue ellipse), and a cassette with the I-SceI gene *SCE1* under the galactose inducible *GAL1-10* promoter, the hygromycin resistance gene *hyg*, as well as the counterselectable *KlURA3* gene in a construct that has been inserted into the *TRP5* gene. FRO-526/527, shown as T5, contains only the *kanMX4* gene and the *KlURA3* gene in a cassette that has been inserted into the *TRP5* gene. All other I-SceI strains shown contain the GSH cassette (the I-SceI gene *SCE1* under the galactose inducible *GAL1-10* promoter and the *hyg* gene) either with the I-SceI break site (T1B, A2B, L2B) shown as a blue ellipse or without the I-SceI site (T1, A2, and L2). Strain HK-225/226, shown as T5B(HO), contains the HO break site (orange ellipse with HO) inserted into the *TRP5* gene.

For the *TRP5* locus, yeast strains FRO-155 (T5B) and FRO-526 (T5) (3) were used (Figure 1). Yeast haploid strain FRO-155 (*MAT*α *his3Δ1 leu2Δ0 lys2Δ0 trp5*::GSHU *lys2*::Alu IR) contains the GSHU cassette (including the I-SceI gene *SCE1* under the inducible *GAL1-10* promoter, the hygromycin resistance gene *hyg* and the counterselectable *URA3* gene from *Kluyveromyces lactis* (*KlURA3* marker gene) and the I-SceI site (HOT site) in *TRP5* (3). FRO-526 is identical to FRO-155 except that instead of the GSHU cassette, the *TRP5* gene is disrupted with the CORE-UK cassette (the counterselectable *KlURA3* marker gene along with the *KanMX4* gene conferring G418 resistance). These strains, along with isogenic isolates FRO-156 (isogenic to FRO-155), and FRO-527 (isogenic to FRO-526) were used for gene correction at the *TRP5* locus. Additionally, *RAD52* deletion in FRO-155 was achieved by replacing the *RAD52* gene with the *kanMX4* gene in strains PAT-44 and PAT-45.

All other strains expressing I-SceI were generated by integrating the GSH cassette (including the I-SceI gene *SCE1* under the inducible *GAL1-10* promoter and the hygromycin resistance gene *hyg*) into each respective locus and strain background. For the strains to contain the I-SceI recognition site [T1B in which GSH with the I-SceI site was integrated into *TRP1* (PAT-18, 19), A2B in which GSH with the I-SceI site was integrated into *ADE2* (PAT-22, 23, 32, 33) and L2B in which GSH with the I-SceI site was integrated into *LEU2* (BPL-1, 2, PAT-20, 21, 34–37)], the GSH cassette was PCR-amplified from plasmid pGSHU (3) using primers with 50-base homology tails to the respective integration site along with the 18-base I-SceI site upstream of the *GAL1-10* promoter (Figure 1). The strains lacking the I-SceI site [T1 in which GSH without the I-SceI site was integrated into *TRP1* (PAT-24, 25), A2 in which GSH without the I-SceI site was integrated into *ADE2* (PAT-28, 29, 42, 43) and L2 in which GSH without the I-SceI site was integrated into *LEU2* (BPL-4, 5, PAT-26, 27, 38–41)] were generated in the same way except with primers lacking the I-SceI site (Figure 1).

Strains HK-225 and HK-226 (isogenic to each other) derive from FRO-767 and were constructed as follows. First, the HO site in *leu2* was eliminated by replacing *leu2*::*HOcs* with an insertion (Supplementary Table S2). Second, a functional *TRP1* gene was introduced in place of *HIS3*, and using the *delitto perfetto* approach (25), the HO cut site (124 bp) was inserted in the middle of *TRP5* exactly in the same position in which we had inserted the I-SceI site in the above described constructs. The sequence of the HO cut site was verified by sequence analysis.

A detailed list of the strains is presented in Supplementary Table S2.

### Yeast transformations using oligonucleotides

Transformations were done as previously described with minor variations (25). Briefly, 50 ml of yeast extract-peptone-lactic acid (YPLac) (lactic acid 2%), pH 5.8, or yeast extract-peptone-dextrose (YPD) liquid culture was inoculated ∼24 h before transformation and incubated with vigorous shaking at 30°C. YPLac was used as a neutral carbon source so as not to induce I-SceI expression until plating the transformations to selective galactose-containing media. YPD was used to repress I-SceI expression until plating the transformations to selective glucose-containing media. Transformations were done with 1 nmol of total oligonucleotide DNA, with the exception of the PAGE-purified oligonucleotides in which transformations were done with 500 pmol of total oligonucleotide DNA. For the transformations testing to determine whether the aptamer was working in *trans*, we used two oligonucleotides, 500 pmol of each oligonucleotide, such that the combined total oligonucleotide DNA used was 1 nmol. Sequences of oligonucleotides used for repair can be found in Supplementary Table S1. Cells from each transformation were diluted appropriately and plated to synthetic complete medium lacking the respective amino acid and containing 2% galactose for I-SceI induction, 2 or 0.2% galactose for HO induction or 2% glucose for I-SceI or HO repression. The exception to this is the experiment to determine the position of the aptamer in the targeting DNA sequence (at the 5′ or 3′ end). For this experiment, I-SceI was induced 3 h by addition of galactose (final concentration 2%) to the liquid YPLac medium before transformation by oligonucleotides, and cells were plated on selective media containing glucose. Viability after transformation was calculated by plating to glucose-containing synthetic complete medium. Viability after transformation for the I-SceI-containing strains was typically 20–40% both on glucose and galactose. The HO-containing strain had a low viability after HO DSB induction, 0.53% in 2% galactose and 1.09% in 0.2% galactose, whereas its viability was ∼25% in glucose. The frequency of gene correction is based on the number of transformants relative to 10^7^ viable cells that formed a colony on galactose-containing synthetic complete medium (or glucose-containing synthetic complete medium depending on the transformation experiment). Yeast cell culture and standard molecular biology techniques were used as previously described (24,26).

### Human cell lines, plasmids and procedures

Human embryonic kidney (HEK-293) cells were grown in Dulbecco’s modified Eagle’s medium (DMEM) (Mediatech, Manassas, VA, USA), supplemented with 10% heat-inactivated fetal bovine serum (Gemini Bio-Products, West Sacramento, CA, USA) and 1× penicillin/streptomycin (Lonza, Walkersville, MD, USA). Cells were grown at 37°C in a water-jacketed 5% CO_2_ humidified incubator (NuAire, Plymouth, MN, USA). Plasmid pLDSLm contains the DsRed2 gene, responsible for producing red fluorescent protein (RFP), disrupted by a 37-bp region containing the 18-bp site for the I-SceI endonuclease preceded by two stop codons. Plasmid pEGFP300-disDsRed2 was modified to make plasmid pLDSLm. pEGFP300-disDsRed2 is identical to pdisEGFP300-disDsRed2 described in Katz *et al.* (27) with the disrupted non-functional GFP replaced by a functional GFP gene. Although not relevant for this work, plasmid pEGFP300-disDsRed2 also contains a LexA DNA binding domain (DBD) site upstream of the CMV promoter of the disrupted DsRed2 gene (inserted using primers LexAF ACAGTGCTAAGTGGATCCGTACTGTATGTACATACAGTACACCGTATTACCGCCATGCAT and LexAR ATTGAGTTCCTAGGATCCGTACTGTATGTACATACAGTACATCTCGGTCTATTCTTTTGA) and a mutated LexA DBD site downstream of the disrupted DsRed2 gene’s polyA tail (inserted using primers IVMLexAM-F CAAAAGAATAGACCGAGATGTACTGTACATATGTACAGTACGGATCTGGTACCTTGTATTA and IVMLexAM-R TAATACAAGGTACC AGATCCGTACTGTACATATGTACAGTACATCTCGGTCTATTCTTTTG). Plasmid pSce (a gift from M. Porteus, Stanford University, CA, USA) contains the I-SceI endonuclease gene expressed under the CMV/CBA promoter as described previously (28). Cells were transfected using polyethylenimine (PEI, Polysciences, Warrington, PA, USA) transfection reagent in 24-well plates seeded at a density of ∼150 000 cells per well (29,30) 24 h before transfection. For transfections in HEK-293 cells, the plasmid DNA was used in the amount of 0.5 µg for the expression vector, as well as 0.5 µg for the targeted vector, and the repairing DNA oligonucleotide used was 1 µg, unless otherwise indicated. For the transfections with I-SceI digested pLDSLm, 0.5 µg of the linearized pLDSLm vector was used with 1 µg of repairing DNA oligonucleotide. Digestion of the pLDSLm vector was done using I-SceI (New England Biolabs, Ipswich, MA, USA). Ten micrograms of plasmid pLDSLm was digested using 15 U I-SceI, 250 ng bovine serum albumin (BSA), 10× I-SceI buffer (New England Biolabs, Ipswich, MA, USA) and water to a final volume of 50 µl. Digestions were done overnight at 37°C. In all transfection experiments, the oligonucleotides and the plasmid were diluted in DMEM without supplements, and then PEI was added, the solution was vortexed and added to the wells 10–15 min later. Red fluorescent cells were visualized by fluorescent microscopy using a Zeiss Observer A1 microscope and an AxioCam MRm camera (Zeiss, Thornwood, NY, USA). Frequencies of RFP positive cells were obtained by flow cytometric analysis using the BD FACS Aria II Cell Sorter (BD Biosciences, Sparks, MD, USA) for RFP detection 5–8 days following transfection. For certain transfections, wells of 24-well plates were seeded with 150 000 cells (on the day before transfection) and 5–8 days after transfection individual fluorescent cells per well were counted using a fluorescence microscope just before flow cytometry analysis. From the seeding time and the time of counting or flowcytometric analysis, cells are 8–10 times more numerous per well. Sequences of oligonucleotides used to repair the DsRed2 gene are listed in Supplementary Table S1.

### mfold secondary structure prediction

Secondary structure prediction software was used on the A7 aptamer-containing oligonucleotides used for gene correction. The program mfold (http://mfold.rna.albany.edu/?q=mfold/DNA-Folding-Form) was used to identify secondary structure at approximately physiological ion concentrations that were also used for the aptamer selection (90 mM Na^+^ and 5 mM Mg^+2^). Folding was done at either 30°C (for the yeast oligonucleotides) or 37°C (for the human oligonucleotides) (31,32).

### Data presentation and statistics

Graphs were made using GraphPad Prism 5 (Graphpad Software, La Jolla, CA, USA). Data are plotted as mean values with 95% confidence intervals shown. Statistical significance was determined using nonparametric two-tailed *t*-tests (Mann–Whitney *U* test).

## RESULTS AND DISCUSSION

### Rationale

This work is designed as a proof-of-principle concept or prototype to show that by binding a protein that guides the correcting donor DNA into proximity to its genetic target via an aptamer, AGT, the efficiency of gene targeting increases. In this study, the chosen protein is the site-specific meganuclease I-SceI. The strategy involves using a DNA aptamer to I-SceI to link the targeting DNA and I-SceI. The hypothesis is that by delivering targeting DNA within proximity to the site of cleavage, gene targeting frequencies can be increased.

### Selection for an aptamer to I-SceI

A fluorescently-labelled DNA library consisting of single-stranded 74-mer oligonucleotides containing a central 36-nt variable region (Supplementary Table S1) was loaded with I-SceI protein on CE with LIF. Two rounds of selection were done to obtain DNA aptamers to I-SceI. Several potential aptamers from the selection pool of aptamers were again run with I-SceI and weak, moderate and strong binding aptamers were identified (Supplementary Figure S3). The 17 strongest binding aptamers identified were selected for sequencing. Several of the sequences were repeated, such that there were only 11 unique strong binding aptamer sequences (Table 1), which showed no general consensus sequence. Of the 11 unique sequences, only three contained the original length of the aptamer region, 36 bases, from the random DNA library. The difference in sequence length from the starting library is not uncommon in aptamer selection (33–35).

**Table 1.** Aptamer sequences to I-SceI

Name	Size	Sequence
A1	17	TCAGTTCCTTGGTTAGG
A3	36	TCTAAGACTTGTGAGTCATACGGTGGGACGCGGTAA
**A4**	28	**TGAAGGCCAAAACGGCTGAATCGATAGT**
A5	26	GCCTTGCTTGAACTGGTAGCACATGT
A6	42	CTCCTGGTCTAGACGAGCCTCACTTTCCAAATCATGACGAGA TAGGCGGAC
**A7**	36	**GCGGGCGCTGTTGACAGCGGTCAGGTGGATGGGATG**
A8	35	CTGCATTTCCTATGGACACAGTGCTTCGTTCAATC
A9	35	GAGTGCCGCGGGGGACTGTCAAGTCGCTGGGTCTA
A10	35	AGGCAGACGCCTCTGACGCAAGGTGCATTGCCTTT
A11	18	ATGTGTATTTGCCAGTAA
A18	36	GTTGCGCTCTAGCTGATCGTGTTTATCCCAAAGGCA

Shown are the 11 strongest I-SceI aptamer sequences. The aptamer A4 and A7 chosen for further analysis are shown in bold. The A7 aptamer is also underlined.

After sequencing the 11 strongest binding aptamers, these candidate aptamers were further characterized using EMSA gels to confirm their binding capacity to I-SceI. Of the candidate aptamers, several showed consistent and reproducible binding to I-SceI (Supplementary Figure S4). Of these, two sequences were chosen to design synthetic oligonucleotides based on their length and their ability to bind to I-SceI, namely, I-SceI aptamer 4 (A4), which was 28 nucleotides, and I-SceI aptamer 7 (A7), which was 36 nucleotides. These two oligonucleotides were synthesized polyacrylamide gel electrophoresis (PAGE) purified, FAM-labelled for fluorescence and underwent further testing by CE with LIF. The binding affinities by CE were calculated to be ∼3.16 µM for A7 (Supplementary Figure S2D) and ∼52.49 µM for A4 (not shown), by a method described previously (36); thus, it appeared that A7 was a stronger binder to I-SceI than A4.

### The I-SceI aptamer stimulates gene correction in yeast

**Figure 2.**
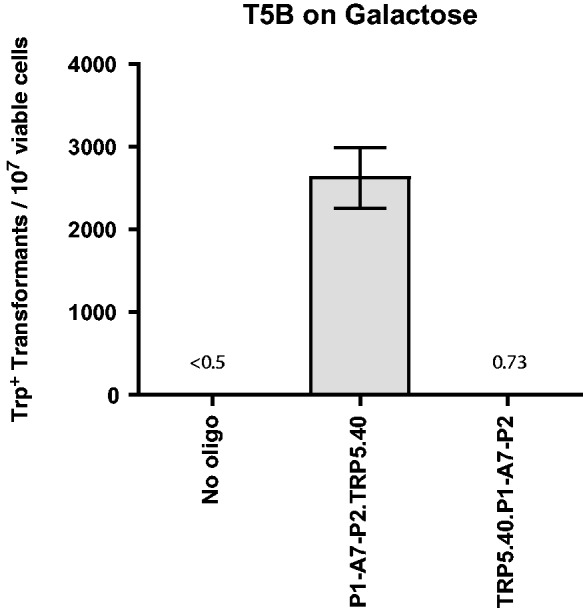
A bifunctional oligonucleotide with the I-SceI aptamer sequence at the 5′ end and the donor sequence at 3′ end is more effective at gene targeting in yeast. Frequency of gene correction in yeast by the A7 aptamer with primers from the random DNA library at either the 5′ or 3′ end of a longer oligonucleotide to repair the *trp5* gene (X axis) measured by the number of Trp^+^ transformants per 10^7^ viable cells (Y axis) in the FRO-155 (T5B) strain plated to galactose media. Bars correspond to the mean value, and error bars represent 95% confidence intervals. For additional information see Supplementary Table S3.

Experiments of gene correction were done in yeast *S. cerevisiae* cells using bifunctional single-stranded DNA oligonucleotides containing the aptamer region of the A4 or A7 I-SceI aptamer at one end, and the donor repairing sequence at the other end. First, we determined on which end (5′ or 3′) the I-SceI aptamer should be positioned in the bifunctional molecule to obtain more effective stimulation of gene targeting. For this experiment, the A7 aptamer with primers P1 and P2 from the random DNA library was synthesized as part of the 5′ end or the 3′ end of the repairing bifunctional oligonucleotides (P1-A7-P2.TRP5.40 for the aptamer with primers at the 5′ end of the bifunctional oligonucleotide and TRP5.40.P1-A7-P2 for the aptamer at the 3′ end) (Supplementary Table S1), which contained 40 bases of homology to correct a disrupted *TRP5* gene in yeast strain FRO-155/156 (T5B in Figure 1) and restore function of the *TRP5* gene. Results showed that having the aptamer region at the 5′ end of the bifunctional molecule was much more efficient at gene targeting than with the aptamer region at the 3′ end (Figure 2). Likely, having the homology region of the donor sequence at the 3′ end facilitates the homology search to the target locus, rather than being a polarity preference of the annealing protein Rad52 (37). The aptamer-containing oligonucleotide with the primers (P1-A7-P2.TRP5.40) was compared with the non-binding random control with primers (P1-r-P2.TRP5.40), and there was a 1.25-fold (*P* = 0.0008) increase using the aptamer-containing oligonucleotide (data not shown). The low increase in gene targeting for this experiment stems from the fact that I-SceI was induced before plating such that most cells already had the DSB before transformation with the oligonucleotides. For all subsequent experiments, I-SceI was induced by directly plating cells on the selective medium containing galactose.

**Figure 3.**
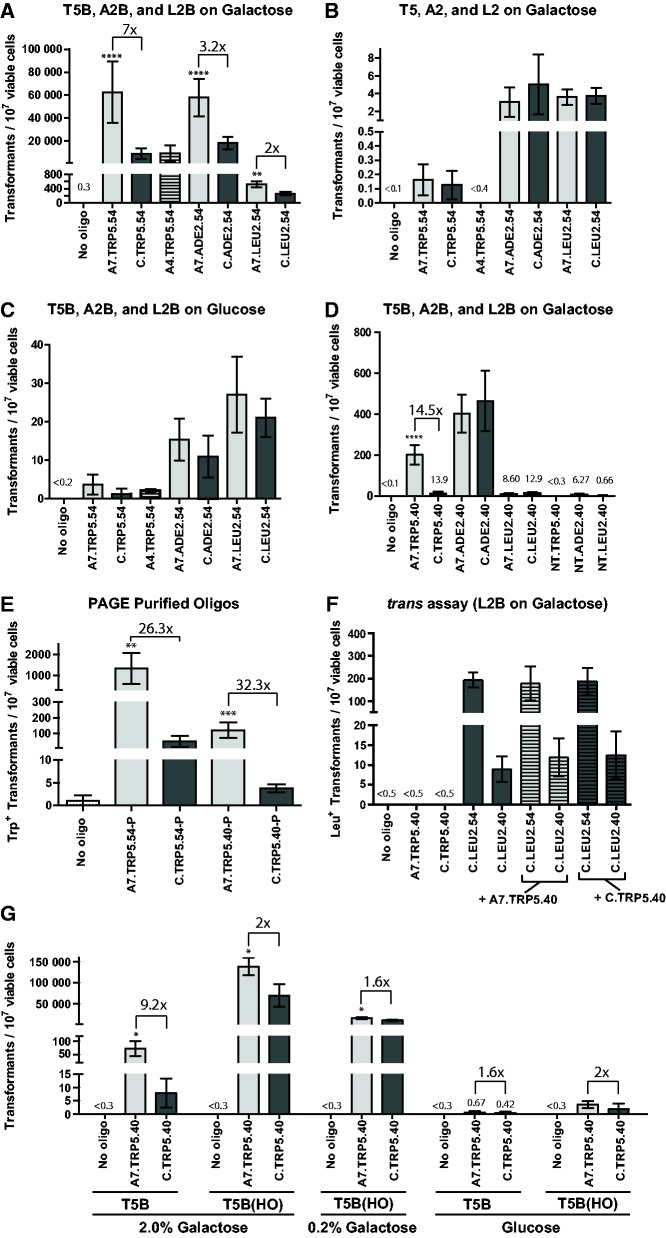
The I-SceI aptamer stimulates gene targeting in yeast strain background BY4742. Frequency of gene correction in yeast using aptamer-containing oligonucleotides shown in light grey and non-binding control oligonucleotides in dark gray (X axis) measured by the number of transformants per 10^7^ viable cells (Y axis) with no oligonucleotide controls averaged in (**A**) strains from the BY4742 background containing the I-SceI break site and the I-SceI gene under the *GAL1-10* promoter grown on galactose media (T5B, A2B and L2B on galactose), (**B**) strains that did not contain the I-SceI site grown on galactose media (T5, A2, and L2 on galactose), (**C**) same strains shown in A) but grown on glucose-containing media (T5B, A2B, and L2B on glucose). (**D**) Frequency of gene correction in yeast by shorter targeting oligonucleotides with only 40 bases of homology, including oligonucleotides without a 5′ non-homologous tail (‘no tail’ NT.TRP5.40, NT.ADE2.40 and NT.LEU2.40) of the aptamer region or the non-binding control sequence. (**E**) Frequency of gene correction in yeast by PAGE-purified oligonucleotides at the *trp5* locus in the FRO-155/156 (T5B) strain grown on galactose for the induction of I-SceI. (**F**) Frequency of gene correction following co-transformation of strains PAT-34 and PAT-35 (L2B), grown on galactose for I-SceI induction, with C.LEU2.54 or C.LEU2.40 with A7.TRP5.40 or C.TRP5.40. (**G**) Frequency of gene correction in yeast by the A7.TRP5.40 or C.TRP5.40 oligonucleotides at the *trp5* locus in the I-SceI containing strain FRO-155/156 (T5B) or in the HO-containing strain HK-225/226 [T5B(HO)] grown on galactose for the induction of I-SceI or HO, or on glucose for the repression of I-SceI or HO. Bars correspond to the mean value and error bars represent 95% confidence intervals. Asterisks denote statistical significant difference between the aptamer-containing oligonucleotide and the corresponding non-binding control (*P < 0.05; **P < 0.01; ***P < 0.001 and ****P < 0.0001), and the fold change in the gene correction frequency is indicated. For additional information see Supplementary Tables S4A and S5–S8.

Previous studies of aptamer selection showed that the primer sequences from the random DNA library that flank the aptamer region in the SELEX process do not generally contribute to aptamer binding (38–40), and they can be removed without affecting the binding function of the aptamer sequence. Therfore, we removed the primer regions surrounding the I-*Sce*I aptamer sequence in the bifunctional oligonucleotides, and this also allowed us to extend the sequence length of the gene-correction donor part of the bifunctional molecule. Oligonucleotides that contained the aptamer sequences from A4 or A7 at the 5′ end of a DNA sequence containing 54 bases of homology to restore the disrupted *trp5* gene (A4.TRP5.54 and A7.TRP5.54, Supplementary Table S1) were tested in yeast strains FRO-155/156 and FRO-526/527 (T5B and T5 in Figure 1) for their capacity to restore the functionality of the *trp5* gene. In addition to the aptamer-containing oligonucleotides used to correct the *trp5* gene a negative control oligonucleotide was used. Because of the inability of the library primers P1 and P2 to bind I-SceI, these were used in place of the aptamer sequence in a new sequence (C) as part of the non-binding negative control oligonucleotide used to repair *trp5* (C.TRP5.54) (Supplementary Table S1). Using the A4 or A7 aptamer-containing oligonucleotides to repair the *trp5* gene, we found that the A7 aptamer-containing oligonucleotide (A7.TRP5.54) significantly increased (*P* < 0.0001) the level of gene correction 7-fold compared with the negative control (C.TRP5.54) or the other aptamer-containing oligonucleotide A4.TRP5.54 (*P* < 0.0001) in the FRO-155/156 strain, in which the I-SceI gene was expressed and the I-SceI site was present at the target site (T5B in Figure 3A). FRO-526, the strain that did not have the I-SceI site and also did not express the I-SceI gene (T5 in Figure 3B), showed no significant difference between the A7 aptamer-containing oligonucleotide A7.TRP5.54, the A4 aptamer-containing oligonucleotide A4.TRP5.54 (*P* = 0.2161) or the negative control C.TRP5.54 (*P* = 0.702). As an additional control, FRO-155/156 (T5B) was grown and plated to glucose-containing media. Without galactose induction for the expression of I-SceI, there was no significant difference between the aptamer-containing oligonucleotide A7.TRP5.54 and the negative control C.TRP5.54 (*P* = 0.814) or the other aptamer-containing oligonucleotide A4.TRP5.54 (*P* = 0.109) (Figure 3C). These controls refute the possibility that the aptamer (A7) sequence is simply protecting the oligonucleotide from degradation better than the non-binding control sequence (C). If the non-binding control sequence did not protect the repair template as effectively as A7, then one would expect that the control sequence would consistently be lower in gene targeting efficiency than the A7 aptamer, which is not the case when the I-SceI site is not present or when the I-SceI protein is not expressed.

### Gene targeting stimulation by the I-SceI aptamer is more effective for a donor with short homology

Although decreasing the donor’s region of homology generally leads to less efficient gene correction (3), we postulated that it might be possible to detect an even greater fold-difference in gene correction efficiency from the aptamer-containing oligonucleotide over the non-binding control if the aptamer was attached to a shorter donor. By shortening the donor homology region of the A7.TRP5.54 bifunctional oligonucleotide from 54 bases to 40 bases (A7.TRP5.40), the overall frequency of repair decreased as expected. However, the frequency of repair at the *trp5* locus using the shorter donor showed an even greater fold difference between the A7 aptamer-containing oligonucleotide and the non-binding negative control (from 7-fold to 15-fold) (*P* < 0.0001) (Figure 3D). Oligonucleotides that contained neither the aptamer region nor the control non-binding sequence [no 5′ non-homologous ‘tail’ (NT in Figure 3D)] were inefficient at targeting (Figure 3D) because without the 5′ non-homologous DNA the oligonucleotide donor sequence was less stable [Storici,F., unpublished data and (24)].Similarly, the overall repair frequency of the A7.TRP5.40 oligonucleotide was lower than that of the P1-A7-P2.TRP5.40 oligonucleotide due to the greater lengths of the P1-A7-P2.TRP5.40 oligonucleotide (Figures 2 and 3D). A possible reason for the increased difference in gene correction frequency between the aptamer-containing molecule and the control molecule could also be that the shorter oligonucleotides were more likely to have the aptamer region intact. Non-purified 100-mer oligonucleotides synthesized at a coupling efficiency of 99.5% contain ∼60% full-length product, with the other 40% being truncated oligonucleotides (41) (truncated at the 5′ end of the oligonucleotide, which in our system would be the aptamer region). Based on the idea that full-length oligonucleotides would have higher likelihood to contain the intact aptamer region, polyacrylamide gel electrophoresis (PAGE)-purified oligonucleotides were tested at the *trp5* locus in yeast strain FRO-155. The PAGE-purified oligonucleotides showed a greater fold difference in repair of *trp5* with the aptamer oligonucleotide as compared with the control than the non-purified oligonucleotides (Figure 3E). Although the fold difference between the aptamer and the control was higher in the purified oligonucleotides [27-fold (*P* = 0.0057) for A7.TRP5.54-P and 32-fold (*P* = 0.0004) for A7.TRP5.40-P], the fold difference of the shorter oligonucleotide (A7.TRP5.40-P) was still more prominent than the longer oligonucleotide (A7.TRP5.54-P). These results suggest the aptamer can be more effective with a shorter homology region.

### Aptamer-guided gene targeting is effective at numerous different targeted genomic loci in yeast

**Figure 4.**
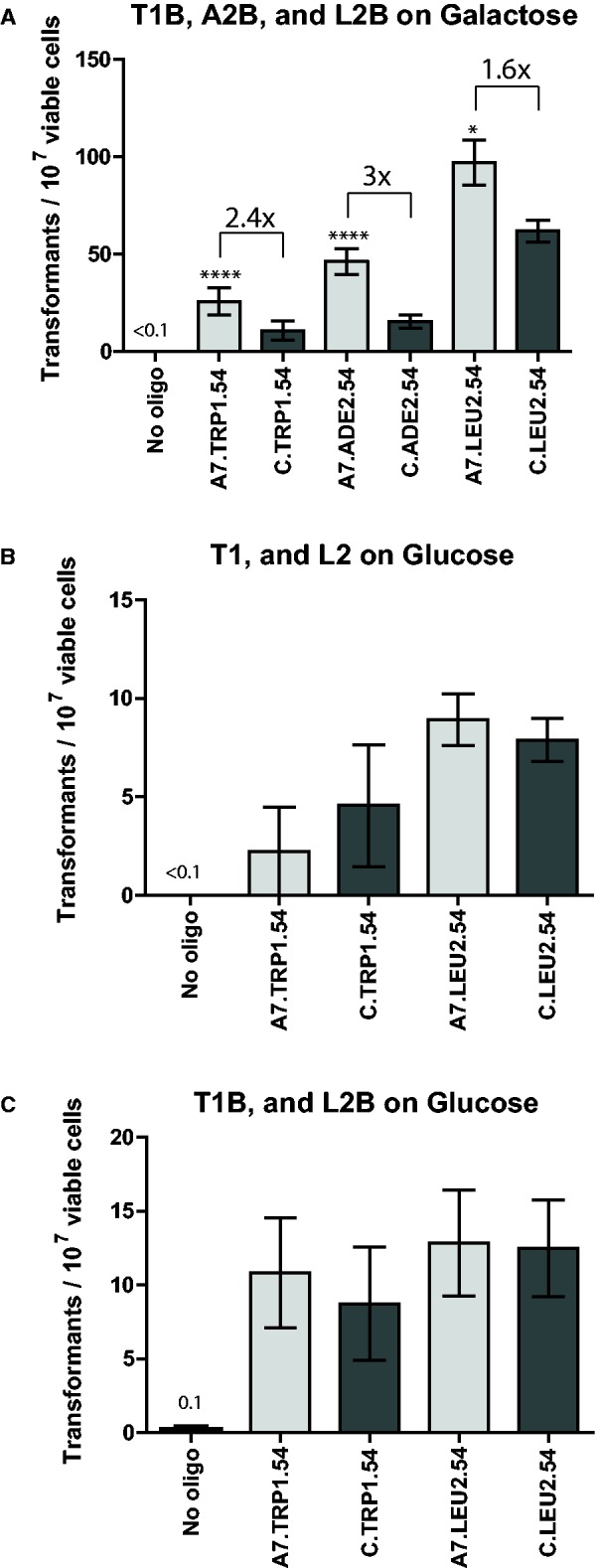
The I-SceI aptamer stimulates gene targeting in yeast strain background 55R5-3C. Frequency of gene correction in yeast using oligonucleotides with aptamer-containing oligonucleotides in light grey and non-binding control oligonucleotides in dark grey (X axis) measured by the number of transformants per 10^7^ viable cells (Y axis) with no oligonucleotide controls averaged in (**A**) strains from the 55R5-3C background containing the I-SceI break site as well as the I-SceI gene under the *GAL1-10* promoter grown on galactose media (T1B, A2B, and L2B on galactose), (**B**) strains that did not contain the I-SceI site grown on glucose containing media (T1, A2, and L2 on glucose), (**C**) same strains shown in A) but grown on glucose-containing media (T1B, A2B or L2B on glucose). For both (B) and (C), the frequency of gene correction for the *ade2* locus was <0.2 and <0.4, respectively (not shown). Bars correspond to the mean value and error bars represent 95% confidence intervals. Asterisks denote statistical significant difference between the aptamer-containing oligonucleotide and the corresponding non-binding control (*P < 0.05; **P < 0.01; ***P < 0.001 and ****P < 0.0001), and the fold change in the gene correction frequency is indicated. For additional information see Supplementary Table S4B.

We next tested the effectiveness of AGT with the I-SceI aptamer at numerous other loci in the yeast genome to verify that the results obtained at the *trp5* gene were not locus specific. In these new loci, the A7 aptamer was compared with the non-binding control (C). Because aptamer A4 was not increasing gene targeting (likely due to its lower binding affinity to I-SceI) at the *trp5* locus, it was not used at other loci. At each of these loci, the GSH cassette containing the I-SceI gene under the inducible *GAL1-10* promoter along with the hygromycin resistance gene *hyg* were integrated into different endogenous marker genes responsible for the metabolism of nucleotides or amino acids, generating auxotrophic mutants for the respective nucleotide or amino acid. The *ADE2* and *LEU2* loci were chosen as targets in the BY4742 strain background, and the *TRP1*, *ADE2* and *LEU2* loci were chosen in the 55R5-3C strain background (Figure 1). For each locus, two types of strains were made in which one had the integrated GSH cassette with the 18-bp I-SceI recognition site (T1B, A2B and L2B in Figure 1) and one strain that had the cassette but did not have the I-SceI site (T1, A2 and L2 in Figure 1) (see ‘Materials and Methods’ section). Following transformation by the bifunctional oligonucleotides with the aptamer for I-SceI or the control region, at every locus tested, using two or more isogenic strain isolates, there was a significant increase in gene targeting with the aptamer-containing (A7) oligonucleotide compared with the negative control (C) oligonucleotide when I-SceI was induced by galactose and the I-SceI site was present (Figures 3A and 4A). There was ∼3-fold increase for the *ade2* locus (*P* < 0.0001 for both BY4742 and 55R5-3C), ∼2-fold increase for the *leu2* locus (*P* = 0.0074 for BY4742 and *P* = 0.0175 for 55R5-3C) and ∼2.5-fold for the *trp1* locus (*P* < 0.0001). We point out that gene correction frequencies in strain 55R5-3C are lower than in BY4742 strain. This could be due to the strain to strain variation because the cassettes used to induce the DSB were the same for the two strains. Despite this difference in the level of correction frequency, the I-SceI aptamer stimulates gene targeting in both strains. Importantly, in the strains lacking the I-SceI site there was no significant difference between the aptamer-containing (A7) and non-binding (C) oligonucleotides (Figures 3B and 4B) (*P* = 1 for the *ade2* locus, and *P* = 0.9297 for the *leu2* locus in the BY4742 background and *P* = 0.185 for the *trp1* locus, *P* = 1 for the *ade2* locus and *P* = 0.5076 for the *leu2* locus in the 55R5-3C background). Likewise, when the strains containing the I-SceI site were grown and plated to glucose-containing media, there was no significant difference between the aptamer-containing (A7) and non-binding (C) oligonucleotides (*P* = 0.4382 for the *ade2* locus, and *P* = 0.1907 for the *leu2* locus in the BY4742 background and *P* = 0.3581 for the *trp1* locus, *P* = 1 for the *ade2* locus and *P* = 0.8252 for the *leu2* locus in the 55R5-3C background) (Figures 3C and 4C).

For the *ade2* and *leu2* loci in the BY4742 strain background by shortening the homology region from 54 to 40 bases, the overall level of repair decreased, similarly to the results at the *trp5* locus. While the shorter aptamer containing oligonucleotide at the *trp5* locus showed a greater fold increase over the non-binding control, no significant difference between the aptamer and the non-binding control oligonucleotides was observed for targeting at the *ade2* or the *leu2* loci using the shorter form of the oligonucleotides (Figure 3D). In addition to shortening the donor length, oligonucleotides were tested in which there was no 5′ non-homologous ‘tail' (NT) that did not contain the aptamer or the non-binding control sequence but only the homology region for gene correction. Each of these oligonucleotides (NT.ADE2.40 and NT.LEU2.40) had very low gene correction frequency (Figure 3D).

### The I-SceI aptamer stimulates gene targeting in *cis*

To exclude the possibility that the aptamer binding to I-SceI could change the structure of I-SceI such that the function of I-SceI would be enhanced, therefore stimulating gene targeting of the donor molecule in *trans*, we performed the following experiment. We transformed the L2B strains in the BY4742 background that contain the I-SceI gene and the I-SceI site with the C.LEU2.54 or the C.LEU2.40 oligonucleotide, each in combination with either the A7.TRP5.40 or the C.TRP5.40 oligonucleotide. Among the aptamer-containing oligonucleotides tested in yeast, the A7.TRP5.40 showed the highest fold-difference over the non-binding control (C.TRP5.40) (Figure 3D). Because both the A7.TRP5.40 and C.TRP5.40 have no capability to repair the *leu2* locus, we envisioned that if the A7.TRP5.40 had stimulated donor targeting in *trans* at the *trp5* locus, A7.TRP5.40 should also stimulate donor targeting at the *leu2* locus by C.LEU2.54 or C.LEU2.40. In this experiment, the C.TRP5.40 would serve as a negative control to A7.TRP5.40 because it does not bind to I-SceI. Co-transformation of C.LEU2.54 or C.LEU2.40 with A7.TRP5.40 did not result in higher frequency of correction than using C.TRP5.40 (*P* = 0.6424 for C.LEU2.54 and *P* = 1 for C.LEU2.40) (Figure 3F). These data support the conclusion that the I-SceI aptamer acts in *cis* with the donor region on the same bifunctional oligonucleotides to stimulate gene targeting. This result also contradicts the possibility that the aptamer binding to I-SceI could be inhibiting I-SceI function to bind or cleave. Aptamer inhibition of DNA binding and/or cleavage by I-SceI would lead to a decrease in gene repair, but cotransformation with A7.TRP5.40 had no effect on *leu2* repair frequency.

### I-SceI aptamer stimulates gene targeting only in the presence of I-SceI and its cut site

To verify the specificity of the I-SceI aptamer, we designed an experiment to test whether the I-SceI aptamer promoted gene targeting only in the presence of I-SceI endonuclease and its cut site or was able to stimulate gene correction also when a different homing endonuclease was expressed and its cut site replaced the I-SceI cut site in the yeast *trp5* locus. We modified a strain (FRO-767) that expresses the HO endonuclease under the *GAL1* inducible promoter and which we previously used to study DSB repair by synthetic oligonucleotides (24). By engineering FRO-767, we constructed strains HK-225 and HK-226, which contain the HO cut site in the middle of the *TRP5* gene in the exact same locus in which the I-SceI site was inserted in our strains [T5B(HO)] (Figure 1). We transformed strains FRO-155/156 (T5B) with the I-SceI endonuclease and its cut site, and HK-225/226 [T5B(HO)] with the HO endonuclease and its cut site using the bifunctional oligonucleotides with the aptamer for I-SceI (A7.TRP5.40) or the control sequence (C.TRP5.40). Cells were incubated in the presence of either 2% galactose to induce the expression of the I-SceI or HO endonuclease, or 2% glucose to repress the expression of the I-SceI or HO endonuclease. For the expression of the HO endonuclease, we also used 0.2% galactose because HO is much more efficient than I-SceI for induction of the DSB [(24) and this work]. Results presented in Figure 3G show that the A7 I-SceI aptamer strongly stimulated gene correction over the control oligonucleotide (9.2-fold by mean comparison; *P* = 0.0286) at the *trp5* locus only with induction of I-SceI by galactose and when the I-SceI cut site was present. Differently, the A7 I-SceI aptamer increased gene correction 1.6–2-fold over the control oligonucleotide with induction of HO by galactose and when the HO cut site was present. Although not statistically significant, a similar 1.6–2-fold effect for the A7 I-SceI aptamer over the control oligonucleotide was also detected in glucose for the I-SceI and HO strains, respectively (Figure 3G and Supplementary Table S8). Thus, while there may be a slight effect by the aptamer alone, it is only the expression of I-SceI, and not HO, that activates the aptamer function of the A7 sequence to stimulate gene targeting by the A7.TRP5.40 oligonucleotide. These results demonstrate that the I-SceI aptamer does not simply stabilize the donor DNA sequence and that most of its stimulatory effect to promote gene correction is specific to I-SceI.

### AGT relies on Rad52

In a r*ad52*-null FRO-155 background strain, repair of *trp5* was much less efficient (Supplementary Figure S5) than in wild-type *RAD52* cells (Figure 3A). Comparing the repair level of A7.TRP5.54 in r*ad52* and *RAD52* cells, there is a significant (*P* < 0.0001) 20 000-fold decrease in the r*ad52*-null background. Similarly, correction frequency by the control C.TRP5.54 oligonucleotide also drops several thousand fold. The r*ad52* strain had a high level of prototrophic clones occurring with no oligonucleotide addition. This is consistent with an increased frequency of large deletions at a DSB site by NHEJ in a r*ad52*-null compared with a wild-type *RAD52* background (42) that after ligation restore the function of the initially disrupted marker gene. Despite the high background level of repair in the r*ad52*-null strain, there was a significant (*P* = 0.0261) difference between the no oligonucleotide control and the aptamer-containing oligonucleotide, but there was no significant difference between the no oligonucleotide control and the non-binding control oligonucleotide (*P* = 0.2432), suggesting that the aptamer may stimulate gene targeting even in the absence of Rad52. However, there was no significant difference between the non-binding control oligonucleotide and the aptamer-containing oligonucleotide (*P* = 0.6902).

### I-SceI aptamer stimulates gene targeting in human cells

In addition to the *in vivo* testing in yeast, we examined the ability of the A7 I-SceI aptamer to stimulate gene targeting in human embryonic kidney (HEK-293) cells. The defective marker gene we used was for the red fluorescent protein (RFP), DsRed2, with the DsRed2 gene carried on a plasmid, pLDSLm. Episomal plasmid substrates are valuable tools to study mechanisms of gene correction in human cells. An I-SceI recognition site and two stop codons disrupted the function of DsRed2. In addition to the targeted plasmid, bifunctional oligonucleotides were transfected along with an I-SceI expression vector, pSce, to repair the DSB generated by the I-SceI nuclease and restore the function of the DsRed2 gene. Similarly to the studies in yeast, we used bifunctional oligonucleotides that contained the A7 aptamer sequence at the 5′ end or a non-binding control sequence of equal length, and a donor sequence of 54 nt with homology to the DsRed2 gene at the 3′ end. As in yeast, the non-binding control consisted of the primers from the random DNA library shown not to influence binding to I-SceI.

**Figure 5.**
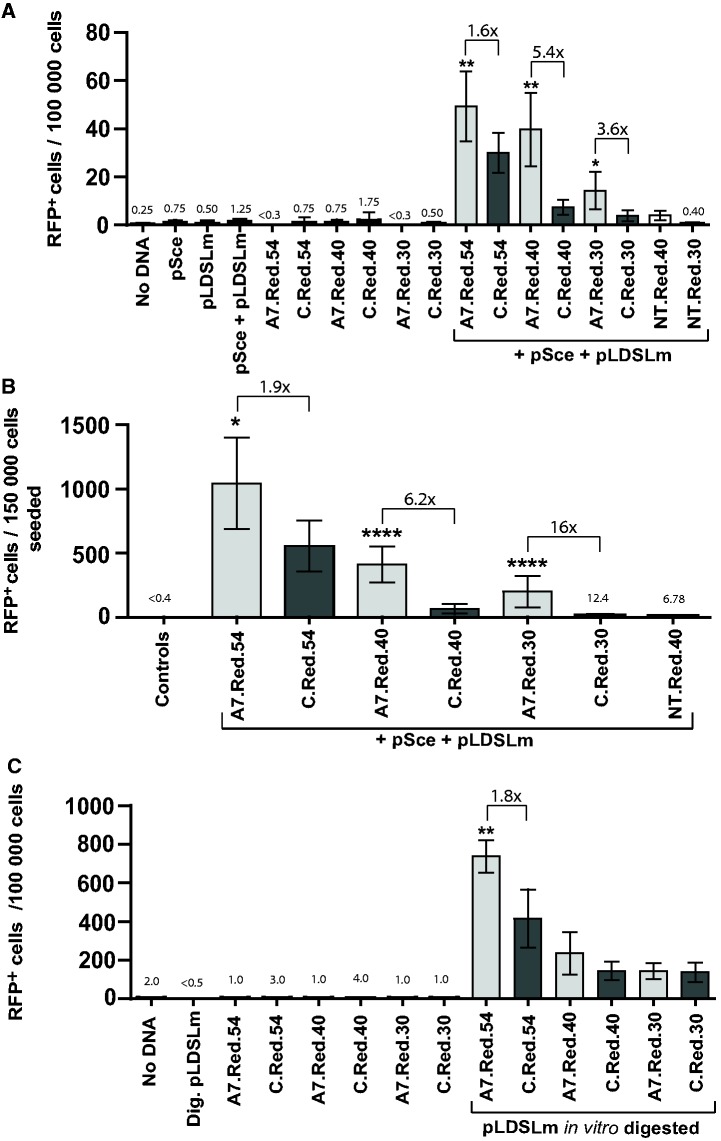
The I-SceI aptamer stimulates gene targeting at the DsRed2 locus in human cells. (**A**) Flow cytometry analysis of several transfections in HEK-293 cells, the different samples are shown on the X axis with aptamer-containing oligonucleotides in light grey and non-binding control oligonucleotides in dark grey and the number of RFP^+^ cells per 100 000 cells is shown on the Y axis. Negative controls were the cells alone (no DNA, only transfection reagent alone), the I-SceI expression vector alone (pSce), the targeted vector (pLDSLm) that contained the DsRed2 gene disrupted with two stop codons and the I-SceI site alone and the individual oligonucleotides alone. Transfections of oligonucleotides with both pSce and pLDSLm added are bracketed. (**B**) Hand counts of each transfection were done in HEK-293 cells in lieu of flow cytometry, which was overreporting the number of background RFP^+^ cells for the shorter oligonucleotides. The different samples are shown on the X axis and the number of RFP^+^ cells per 150 000 cells seeded is shown on the Y axis. Negative controls did not show any RFP^+^ cells. (**C**) Flow cytometry analysis of transfections of the *in vitro* digested pLDSLm vector, the different samples shown on the X axis and the number of RFP^+^ cells per 100 000 cells is shown on the Y axis. Negative controls were the cells alone (no DNA), the digested vector alone and the individual oligonucleotides alone. Transfections with both the digested vector and an oligonucleotide are bracketed. Bars correspond to the mean value and error bars represent 95% confidence intervals. Asterisks denote statistical significant difference between the aptamer-containing oligonucleotide and the corresponding non-binding control (*P < 0.05; **P < 0.01; ***P < 0.001 and ****P < 0.0001), and the fold change in the gene correction frequency is indicated. For additional information see Supplementary Tables S9 and S10.

Using an oligonucleotide containing the A7 aptamer and 54 bases of homology to DsRed2 (A7.Red.54), there was a significant (*P* = 0.0012), ∼2-fold, increase in repair over the non-binding control (Figure 5A). As in yeast, oligonucleotides with shorter homology regions 3′ to the aptamer or non-binding control sequence were designed and tested, using oligonucleotides with 40 or 30 bases of homology (Supplementary Table S1). Similar to our results at the *TRP5* locus in yeast, the shorter oligonucleotides with 40 bases of homology (A7.Red.40 and C.Red.40) had lower overall gene targeting frequency compared with the longer oligonucleotides (A7.Red.54 and C.Red.54) due to the decreased homology of these oligonucleotides. However, comparing repair of the A7.Red.40 oligonucleotide relative with the corresponding non-binding control oligonucleotide C.Red.40 (Figure 5A), there was a 6-fold increase (*P* = 0.0067) in gene targeting measured by flow cytometry. Comparing the A7.Red.30 and C.Red.30 oligonucleotides by flow cytometry, there was a 4-fold increase (*P* = 0.0146). We observed a high level of background from the flow cytometer that we thought could be obscuring the fold-difference seen with the aptamer-containing oligonucleotides over the non-binding control oligonucleotides, especially in the case of the shortest oligonucleotides, those with only 30 bases of homology to DsRed2. Using fluorescence microscopy, manual hand counts of the RFP^+^ cells in each well were conducted (Figure 5B), and for the oligonucleotides with 54 and 40 bases of homology, the hand counts and the readings by flow cytometry were in agreement, but for the A7.Red.30 oligonucleotide compared with the C.Red.30 oligonucleotide, a 16-fold increase (*P* < 0.0001) in repair relative to the non-binding control was observed instead of the 4-fold increase detected by flow cytometry.

AGT relies on the presence of the I-SceI protein to drive the aptamer-containing correction oligonucleotide to the targeted site, and without I-SceI expression there was no significant difference between the aptamer-containing oligonucleotides and the non-binding oligonucleotides in yeast. To verify the increase in gene targeting in human cells by AGT, the targeted vector (pLDSLm) was digested with I-SceI *in vitro* before transfection. By digesting the vector *in vitro* and without co-transfection of the I-SceI expression vector, the aptamer would not be able to be targeted to the I-SceI site by I-SceI. Following co-transfection of the linearized vector and the oligonucleotides, the overall frequency of RFP^+^ cells increased for both the aptamer-containing and control oligonucleotides compared with experiments in which the I-SceI DSB was generated *in vivo*. This is expected because the I-SceI site had been efficiently cleaved before transfection by overnight *in vitro* digestion of the pLDSLm vector with excess I-SceI enzyme. Differently from the results obtained in human cells expressing I-SceI that are presented in Figure 5A and B, without the vector expressing I-SceI there was only a 1.6-fold difference (although not statistically significant, *P* = 0.0952) between the A7.Red.40 and the non-binding C.Red.40 control, and no difference (*P* = 1) between the A7.Red.30 and the non-binding C.Red.30 control (Figure 5C). However, for the A7-aptamer containing oligonucleotide with 54 bases of homology to DsRed2 (A7.Red.54) compared with the non-binding control (C.Red.54), there was a significant 1.75-fold difference (*P* = 0.0079). These data demonstrate that the increase in RFP^+^ frequency observed for the shorter A7.Red.40 and A7.Red.30 oligonucleotides (6-fold for A7.Red.40, and 4-fold for A7.Red.30 or 16-fold with the hand counts for the same oligonucleotide) (Figure 5A and B) is due to the A7 aptamer sequence of these oligonucleotides, and it occurs only when the I-SceI protein is expressed in the targeted cells.

### I-SceI aptamer stem-loop secondary structure is important for I-SceI AGT

**Figure 6.**
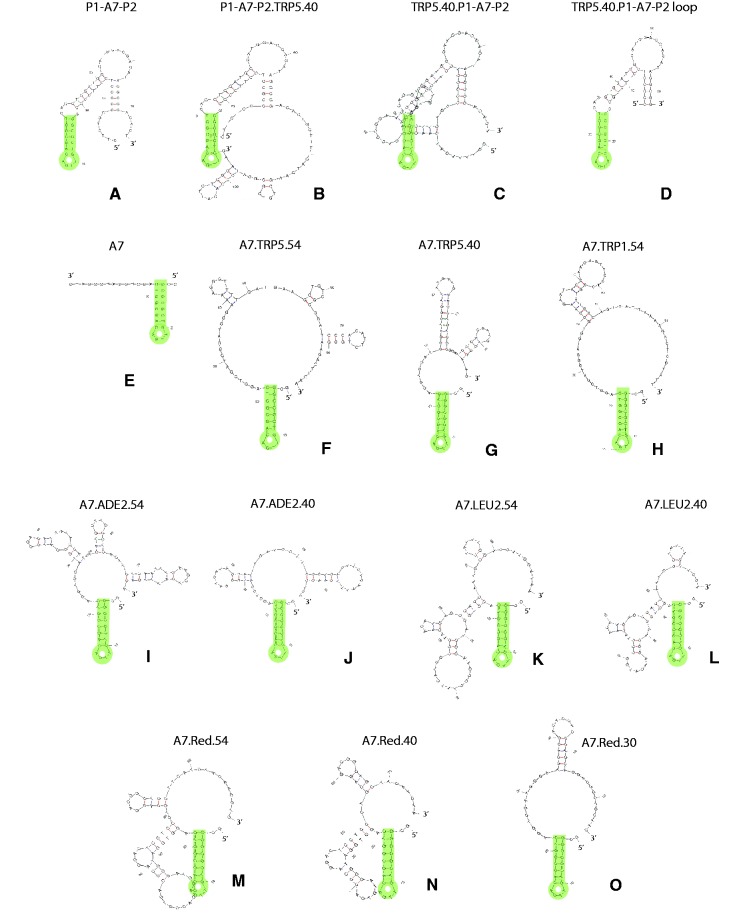
Predicted secondary structure of oligonucleotides containing the I-SceI aptamer. Lowest free-energy secondary structures predicted for the oligonucleotides used in yeast and human cells containing the A7 aptamer sequence (aptamer hairpin highlighted in green). (**A**) The A7 aptamer with primers P1 and P2 from the random DNA library. (**B**) P1-A7-P2.TRP5.40 oligonucleotide. (**C**) The TRP5.40.P1-A7-P2 oligonucleotide. (**D**) The aptamer loop from (C), which was obscured by the overlapping stem-loop. (**E**) The A7 aptamer without primers from the random DNA library. (**F**) The A7.TRP5.54 oligonucleotide. (**G**) The A7.TRP5.40 oligonucleotide. (**H**) The A7.TRP1.54 oligonucleotide. (**I**) The A7.ADE2.54 oligonucleotide. (**J**) The A7.ADE2.40 oligonucleotide. (**K**) The A7.LEU2.54 oligonucleotide. (**L**) The A7.LEU2.40 oligonucleotide. (**M**) The A7.Red.54 oligonucleotide. (**N**) The A7.Red.40 oligonucleotide. (**O**) The A7.Red.30 oligonucleotide.

For the aptamer to bind to I-SceI, it must form a particular structure. The additional DNA needed for gene correction could potentially disrupt the aptamer structure. To investigate this possibility secondary structure prediction software mfold (http://mfold.rna.albany.edu/?q=mfold/dna-folding-form) was used on all the oligonucleotides tested in yeast and human cells for gene correction to determine lowest free energy secondary structures under physiological conditions (see ‘Materials and Methods’ section). Using this program, the A7 aptamer with both primers from the DNA library shows a hairpin with a 4-nt loop that forms from the internal aptamer sequence (Figure 6A). This hairpin was seen in both of the oligonucleotides (P1-A7-P2.TRP5.40 and TRP5.40.P1-A7-P2) used to determine which end (5′ or 3′) of the bifunctional oligonucleotide the aptamer region should be on (Figure 6B–D). The aptamer region without the primers showed a similar hairpin structure at its 5′ end (Figure 6E). The lowest free-energy (most stable) structures predicted for the bifunctional A7 aptamer-containing oligonucleotides in yeast with 54-base homology regions (A7.TRP5.54, A7.TRP1.54, A7.ADE2.54 and A7.LEU2.54) all formed this aptamer hairpin near the 5′ end of the oligonucleotide (Figure 6F, H, I and K). It was interesting to note, however, that while the A7.TRP5.54, A7.TRP1.54 and A7.ADE2.54 oligonucleotides had several bases without secondary structure following the aptamer hairpin on the 3′ side, the oligonucleotide to repair *LEU2* (A7.LEU2.54) contained only a single base between the aptamer hairpin and another stem-loop structure. This might explain why the A7.LEU2.54 oligonucleotide, while still capable of increasing gene targeting, showed the least fold difference in repair over the non-binding control. When analysing the secondary structures of the A7 aptamer-containing oligonucleotides with 40 base homology regions (A7.TRP5.40, A7.ADE2.40 and A7.LEU2.40), there was no significant change in secondary structure for each of the oligonucleotides compared with the longer oligonucleotides, except for the A7.TRP5.40 oligonucleotide, which formed a stable stem loop structure but opposite to the aptamer hairpin (Figure 6G, J and L). Analysing the predicted secondary structure of the bifunctional oligonucleotides to target DsRed2, we found that the DsRed2 aptamer-containing oligonucleotide with 54 bases of homology (A7.Red.54) was predicted to form the aptamer hairpin (Figure 6 M). Similar to the A7.LEU2.54 oligonucleotide, the A7.Red.54 oligonucleotide was predicted to have another large stem-loop structure close to the 3′ end of the aptamer hairpin, and this would be consistent with the *in vivo* result that for the A7.Red.54 oligonucleotide the A7 aptamer is not facilitating gene targeting compared with the non-binding control in human cells via its specific interaction with I-SceI. If the A7 aptamer structure was unable to form for the A7.Red.54 oligonucleotide, it would explain the similar fold-difference over the non-binding control with or without I-SceI expression (2-fold with I-SceI expression and 1.75-fold without). For the shorter oligonucleotides with 40 (A7.Red.40) or 30 (A7.Red.30) bases of homology to DsRed2 (Figure 6N and O), both were predicted to form the aptamer hairpin. The A7.Red.40 oligonucleotide is similar in structure to the A7.Red.54 oligonucleotide, except that the stem-loop predicted to form after the aptamer hairpin has a smaller loop region (7 bases compared with 13 bases), which may not interfere with the aptamer binding. Interestingly, the A7.Red.30 oligonucleotide, which had the highest fold difference compared with the non-binding control (C.Red.30) in human cells, had a secondary structure similar to that of the A7.TRP5.40 oligonucleotide, which showed the highest fold difference in yeast. Taken as a whole, these results provide relevant insights into oligonucleotide design for AGT. In the case of the I-SceI aptamer, the design of oligonucleotides to be most efficient for AGT is the one in which the aptamer stem-loop structure is intact and distant from other secondary structures. Although not employed here, the use of a linker between the aptamer and the homology regions might prove useful to ensure proper binding to I-SceI by the A7 aptamer.

## CONCLUSION

**Figure 7.**
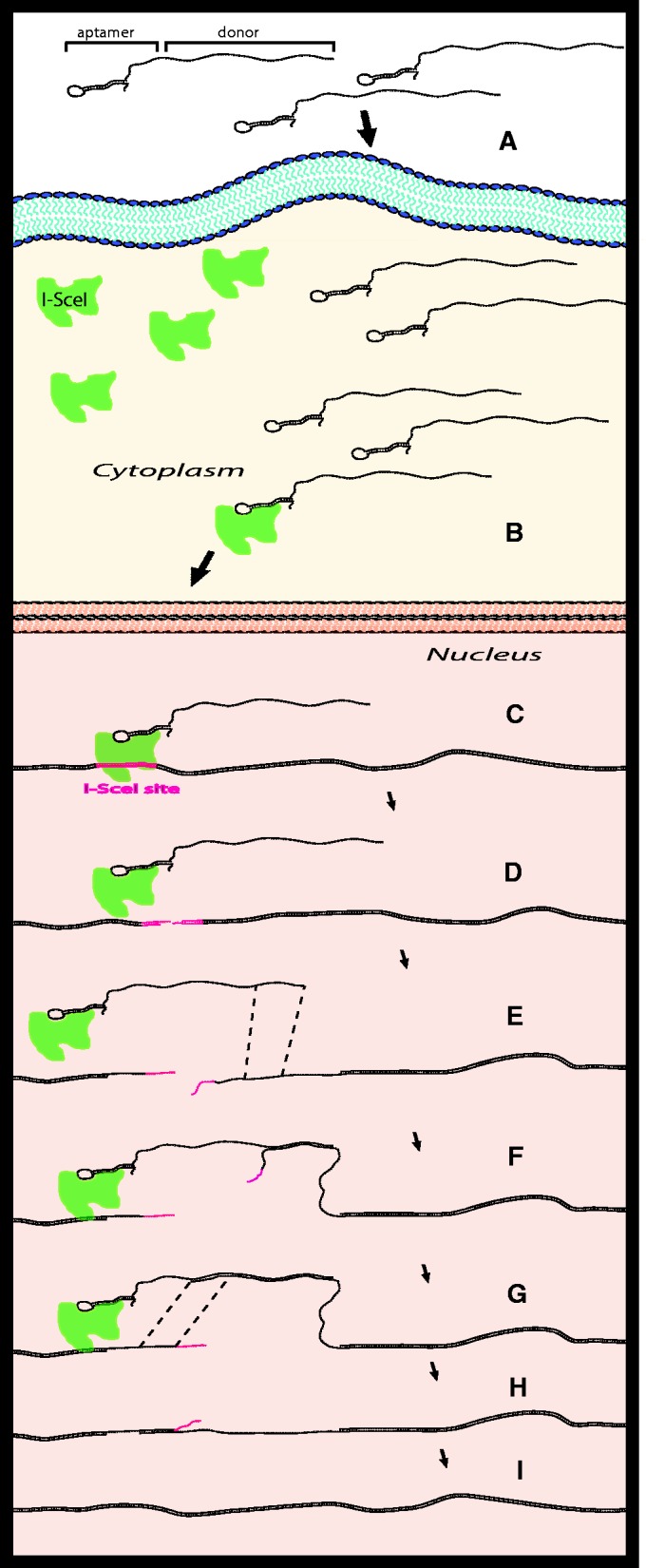
Aptamer-guided gene targeting model. (**A**) Bifunctional-targeting oligonucleotides containing the A7 aptamer at the 5′ end along with a region of homology to restore the function of a defective gene of interest are transformed/transfected into the cell. The I-SceI endonuclease is produced from the chromosome (yeast) or from a transfected expression vector (humans). (**B**) The A7 aptamer then binds to the I-SceI protein, either in the cytoplasm (shown here) or in the nucleus. (**C**) I-SceI drives the bifunctional oligonucleotide to the targeted locus containing the I-SceI site, and (**D**) generates a DSB at the I-SceI site. (**E**) Resection of the 5′ ends of the DSB gives rise to single-stranded 3′ DNA tails. (**F**) The 3′ tail of the bifunctional oligonucleotide anneals to its complementary DNA sequence on the targeted DNA, and after the non-homologous sequence is clipped, (**G**) DNA synthesis proceeds on the template sequence. (**H**) After unwinding of the bifunctional oligonucleotide, a second annealing step occurs between the extended 3′ end and the other 3′ end generated from the DSB. (**I**) Further processing, gap-filling DNA synthesis, and subsequent ligation complete repair and modification of the target locus.

The AGT described here takes advantage of the fact that a single DNA molecule can have more than one function. By constructing a bifunctional DNA oligonucleotide to contain an aptamer region at its 5′ end and a donor region to repair a genomic locus at its 3′ end, we were able to tether the donor DNA of choice to the site-specific endonuclease I-SceI. By tethering the donor DNA to I-SceI, we succeeded in delivering the donor DNA close to the site of the I-SceI DSB, and thus next to the desired targeting locus. Using bifunctional oligonucleotides in which the predicted hairpin structure of the aptamer to I-SceI formed, we stimulated gene targeting specifically when the I-SceI endonuclease was expressed and the I-SceI site was present in every genomic locus tested, in both yeast (up to 32-fold) and human cells (up to 16-fold). Overall, in this study, we provide several lines of evidence that a DNA aptamer for a homing endonuclease, like I-SceI, can guide donor DNA to the vicinity of the nuclease cut site to increase the efficiency of gene correction close to the cut site and enhance the specificity of the genetic modification (Figure 7). Exploiting the I-SceI aptamer in AGT, the donor molecule is brought in the vicinity of its target site, and this may not only increase HR with the desired locus but also potentially reduce the likelihood of random integration.

In addition to the efficacy of the I-SceI aptamer at increasing gene targeting, the AGT system provides a novel function for aptamers. Aptamers themselves are a relatively new discovery, with the first aptamer selection protocols separately published in 1990 (43,44). Aptamers have been used as biosensors (45,46) and as therapeutics (47), but much of their function can be simplified to binding and fluorescing (sensor) or binding and inhibiting (therapy) or binding and being endocytosed (therapy). The aptamer for I-SceI that we selected binds and is targeted to a specific DNA site. This represents not only a novel gene targeting strategy but also a novel use of an aptamer. The novelty and uniqueness of our AGT system lay the foundation for generating and exploiting other aptamers for gene targeting. It may be possible to obtain aptamers for other site-specific homing endonucleases, or for more modular nucleases, like zinc-finger nucleases (ZFNs) (48), transcription activator-like effector nucleases (TALENs) (49) or the Cas9 nuclease of the clustered regularly interspaced short palindromic repeat (CRISPR) system (50). Furthermore, the potential aptamer targets to stimulate gene correction in cells are not limited to endonucleases but could be any protein that facilitates the targeting process, such as transcription factors, HR proteins or even NHEJ proteins. With our AGT system, there was a significant increase in gene repair, and it is possible that stronger binding aptamers would have an even greater impact. The work described here represents a novel proof-of-principle study, where we show that aptamers can be used as tools for gene targeting.

## SUPPLEMENTARY DATA

Supplementary Data are available at NAR Online.

## FUNDING

This work was supported by Georgia Cancer Coalition [R9028]; NIH [R21EB9228]; Georgia Tech Fund for Innovation in Research and Education [GT-FIRE-1021763 to F.S.]. Funding for open access charge: Georgia Institute of Technology (to F.S.).

*Conflict of interest statement*. None declared.

## Supplementary Material

Supplementary Data
