# Supplementary material for: Aptamer-guided gene targeting in yeast and human cells
Source: Nucleic Acids Res. 2014 Feb 5;42(7):e61. doi: 10.1093/nar/gku101 (PMC3985672; doi:10.1093/nar/gku101)
Supplement: Supplementary Data [file supp_42_7_e61__index.html]

Aptamer-guided gene targeting in yeast and human cells — Aptamer-guided gene targeting in yeast and human cells — Supplementary Data 

# Aptamer-guided gene targeting in yeast and human cells

## Supplementary Data

files

**Files in this Data Supplement:**

- Supplementary Data - pdf file
