# Supplementary material for: Aptamer-guided gene targeting in yeast and human cells
Source: Nucleic Acids Res. 2014 Feb 5;42(7):e61. doi: 10.1093/nar/gku101 (PMC3985672; doi:10.1093/nar/gku101)

SUPPLEMENTARY MATERIALS AND METHODS

Electrophoretic Mobility Shift Assay (EMSA)

Potential aptamer oligonucleotides and a negative control oligonucleotide were 5' labeled with P^{32} γ-ATP using T4 Polynucleotide Kinase (New England Biolabs, Ipswich, MA). The negative control (P1-r-P2) consisted of an oligonucleotide of the same length as the random DNA library (74 bases), contained the same flanking primer regions, and had a fixed sequence for its internal region 5'-CTTCTGCCCGCCTCCTTCCGGTCGGGCACACCTGTCATACCC AATCTCGAGGCCAGACGAGATAGGCGGACACT-3' (**Supplementary Table 1**). The internal region was chosen using a random DNA sequence generator with a specified GC content of 50% (http://www.faculty.ucr.edu/~mmaduro/random.htm). I-SceI was dialyzed before running the EMSA gels in Run Buffer 1 (RB1), 50mM Tris-HCl at pH 8.2, as previously described. Bovine serum albumin (BSA) was purchased as a lyophilized powder through Sigma-Aldrich (St. Louis, MO) and was greater than 98% pure. BSA stock of 10 mg/ml was made in RB1 buffer.

The buffer conditions used for binding had several components described previously (22). Each reaction consisted of 2 µL 5X EMSA buffer 1 (100 mM Tris-HCl at pH 8.5, 250 mM NaCl, 10 µM $ZnCl_2$, 10 mM $MgCl_2$, 10% glycerol), 1 µL BSA (10 mg/ml), 1 µL freshly prepared 20 mM DTT, and 1 µL 100 mM $MgCl_2$ for a final buffer concentration of 20 mM Tris-HCl, 50 mM NaCl, 2 µM $ZnCl_2$, 22 mM $MgCl_2$, 1mg/ml BSA, 24 mM DTT, and 2% glycerol. After mixing these components together, 2 µL of dialyzed I-SceI (total of 3 µM) for each reaction was added, bringing the volume to 7 µL. The radiolabeling of the oligonucleotides had varying efficiencies such that the counts per minute (cpm) measurement of the radioactivity for each labeled oligonucleotide was different. Reactions were aliquoted and 0.3 to 0.5 µl (~20,000 cpm equivalent) of γP^{32}-labeled oligonucleotides were added. The reaction mixture of DNA and I-SceI was incubated for 70 minutes at room temperature. After incubation 2 µl of EMSA buffer 2 (120 mM Tris-HCl at pH 8, 600 mM NH_4Cl, 300 mM NaCl, 300 mM KCl, 30% glycerol, 0.25% bromophenol blue) was added. After addition of EMSA buffer 2 the samples were iced until loaded. The reactions were run on 4% polyacrylamide gels under non-denaturing conditions. Mini-gels were made with stock solutions of 40% acrylamide/bis-acrylamide (29:1), 1X Tris-borate EDTA (TBE), 10% ammonium persulfate (APS), and tetramethylethylenediamine (TEMED). Gels were run using the Mini-PROTEAN Tetra Cell apparatus from BioRad (Hercules, CA). Pre-run was done in 1X TBE buffer for 1 hour prior to loading of the samples. The samples were run in cold running buffer at 150 V until the bromophenol blue dye reached the bottom of the gel. The radioactivity in the gel was analyzed by Phosphor Imager (Molecular Dynamics – Typhoon Trio Imager, GE Healthcare Life Sciences, Pittsburgh, PA). The imager was set to highlight saturated pixels. Due to the single-stranded DNA used, aggregates likely formed that were unable to enter the gel and these aggregates were reduced in the presence of protein.

Supplementary Table 1 Oligonucleotides used in this study

Name	Size	Sequence
**Selection Oligos**		
Library	74	5’ **FAM-**CTTCTGCCCGCCTCCTTCCNNNNNNNNNNNNNNNNNNNNNNNNN NNNNNNNNNNNGACGAGATAGGCGGACACT 3’
P1	19	5’ CTTCTGCCCGCCTCCTTCC 3’
P2	19	5’ AGTGTCCGCCTATCTCGTC 3’
P1-FAM	19	5’ **FAM-**CTTCTGCCCGCCTCCTTCC 3’
P1-r-P2	74	5’ CTTCTGCCCGCCTCCTTCCGGTCGGGCACACCTGTCATACCCAATCTCGAGG CCAGACGAGATAGGCGGACACT 3’
**Yeast Oligos**		
P1-A7-P2.TRP5.40	114	5’ CTTCTGCCCGCCTCCTTCC GCGGGCGCTGTTGACAGCGGTCAGGTGGATGGGATG GACGAGATAGGCGGACACTGGTTTTGATGAAGCTGTCGCGGATCCCACATTCTGGGA AG 3’
TRP5.40.P1-A7-P2	114	5’ GGTTTTGATGAAGCTGTCGCGGATCCCACATTCTGGGAAGCTTCTGCCCGCCT CCTTCC GCGGGCGCTGTTGACAGCGGTCAGGTGGATGGGATGGACGAGATAGGC GGACACT 3’
P1-r-P2.TRP5.40	114	5’ CTTCTGCCCGCCTCCTTCCGGTCGGGCACACCTGTCATACCCAATCTCGAGG CCAGACGAGATAGGCGGACACTGGTTTTGATGAAGCTGTCGCGGATCCCACATTCTG GGAAG 3’
A7.TRP5.54	90	5' GCGGGCGCTGTTGACAGCGGTCAGGTGGATGGGATG GGAAAAGGGTTTTGATGAA GCTGTCGCGGATCCCACATTCTGGGAAGACTTCAA 3'
A7.TRP5.54-P	90	5' GCGGGCGCTGTTGACAGCGGTCAGGTGGATGGGATG GGAAAAGGGTTTTGATGAA GCTGTCGCGGATCCCACATTCTGGGAAGACTTCAA 3'
A7.TRP5.40	76	5' GCGGGCGCTGTTGACAGCGGTCAGGTGGATGGGATG GGTTTTGATGAAGCTGTCG CGGATCCCACATTCTGGGAAG 3'
A7.TRP5.40-P	76	5' GCGGGCGCTGTTGACAGCGGTCAGGTGGATGGGATG GGTTTTGATGAAGCTGTCG CGGATCCCACATTCTGGGAAG 3'
A7.TRP1.54	90	5’ GCGGGCGCTGTTGACAGCGGTCAGGTGGATGGGATG GTGGCAAGAATACCAAGA GTTCCTCGGTTTGCCAGTTATTAAAAGACTCGTATT 3’

A7.ADE2.54	90	5’ GCGGGCGCTGTTGACAGCGGTCAGGTGGATGGGATG GGACATTATACCATTGATG CTTGCGTCACTTCTCAATTTGAAGCTCATTTGAGA 3’
A7.ADE2.40	76	5’ GCGGGCGCTGTTGACAGCGGTCAGGTGGATGGGATG ATACCATTGATGCTTGCGT CACTTCTCAATTTGAAGCTCA 3’
A7.LEU2.54	90	5’ GCGGGCGCTGTTGACAGCGGTCAGGTGGATGGGATG CGCTTTCATGGCCCTACAA CATGAGCCACCATTGCCTATTTGGTCCTTGGATAA 3’
A7.LEU2.40	76	5’ GCGGGCGCTGTTGACAGCGGTCAGGTGGATGGGATG ATGGCCCTACAACATGAGC CACCATTGCCTATTTGGTCCT 3’
C.TRP5.54	90	5' TTCTGCCCGCCTCCTTCCGACGAGATAGGCGGACAC GGAAAAGGGTTTTGATGAA GCTGTCGCGGATCCCACATTCTGGGAAGACTTCAA 3'
C.TRP5.54-P	90	5' TTCTGCCCGCCTCCTTCCGACGAGATAGGCGGACAC GGAAAAGGGTTTTGATGAA GCTGTCGCGGATCCCACATTCTGGGAAGACTTCAA 3'
C.TRP5.40	76	5' TTCTGCCCGCCTCCTTCCGACGAGATAGGCGGACAC GGTTTTGATGAAGCTGTCGC GGATCCCACATTCTGGGAAG 3'
C.TRP5.40-P	76	5' TTCTGCCCGCCTCCTTCCGACGAGATAGGCGGACAC GGTTTTGATGAAGCTGTCGC GGATCCCACATTCTGGGAAG 3'
C.TRP1.54	90	5’ TTCTGCCCGCCTCCTTCCGACGAGATAGGCGGACAC GTGGCAAGAATACCAAGAGTTCCTCGGTTTGCCAGTTATTAAAAGACTCGTATT 3’
C.ADE2.54	90	5’ TTCTGCCCGCCTCCTTCCGACGAGATAGGCGGACAC CGCTTTCATGGCCCTACAACATGAGCCACCATTGCCTATTTGGTCCTTGGATAA 3’
C.ADE2.40	76	5’ TTCTGCCCGCCTCCTTCCGACGAGATAGGCGGACAC ATGGCCCTACAACATGAGCCACCATTGCCTATTTGGTCCT 3’
C.LEU2.54	90	5’ TTCTGCCCGCCTCCTTCCGACGAGATAGGCGGACAC CGCTTTCATGGCCCTACAACATGAGCCACCATTGCCTATTTGGTCCTTGGATAA 3’
C.LEU2.40	76	5’ TTCTGCCCGCCTCCTTCCGACGAGATAGGCGGACAC ATGGCCCTACAACATGAGCCACCATTGCCTATTTGGTCCT 3’
A4.TRP5.54	90	5' TGAAGGCCAAAACGGCTGAATCGATAGTGGAAAAGGGTTTTGATGAA GCTGTCGCGGATCCCACATTCTGGGAAGACTTCAA 3'
NT.TRP5.40	40	5’ GGTTTTGATGAAGCTGTCGCGGATCCCACATTCTGGGAAG 3’
NT.ADE2.40	40	5’ ATGGCCCTACAACATGAGCCACCATTGCCTATTTGGTCCT 3’
NT.LEU2.40	40	5’ ATGGCCCTACAACATGAGCCACCATTGCCTATTTGGTCCT 3’
**Mammalian Oligos**		

A7.Red.54	90	5’GCGGGCGCTGTTGACAGCGGTCAGGTGGATGGGATGGCGACCGTGACCCAGGACT CCTCCCTGCAGGACGGCTGCTTCATCTACAAGGTG 3’
A7.Red.40	76	5’GCGGGCGCTGTTGACAGCGGTCAGGTGGATGGGATGTGACCCAGGACTCCTCCCTG CAGGACGGCTGCTTCATCTA 3’
A7.Red.30	66	5’GCGGGCGCTGTTGACAGCGGTCAGGTGGATGGGATGCAGGACTCCTCCCTGCAGG ACGGCTGCTTC 3’
C.Red.54	90	5' TTCTGCCCGCCTCCTTCCGACGAGATAGGCGGACAC GCGACCGTGACCCAGGACTCCTCCCTGCAGGACGGCTGCTTCATCTACAAGGTG 3'
C.Red.40	76	5' TTCTGCCCGCCTCCTTCCGACGAGATAGGCGGACACTGACCCAGGACTCCTC CCTGCAGGACGGCTGCTTCATCTA 3'
C.Red.30	66	5' TTCTGCCCGCCTCCTTCCGACGAGATAGGCGGACACCAGGACTCCTCCCTGCA GGACGGCTGCTTC 3'
NT.Red.40	40	5’ TGACCCAGGACTCCTCCCTGCAGGACGGCTGCTTCATCTA 3’
NT.Red.30	30	5’ CAGGACTCCTCCCTGCAGGACGGCTGCTTC 3’

Shown are the oligonucleotides used in the aptamer selection, yeast experiments, and mammalian experiments. The aptamer sequences are underlined.

Supplementary Table 2 Strains used for yeast studies

Strain Name (nickname)	Relevant Genotype	Source
BY4742	*MATα his3Δ1 leu2Δ0 lys2Δ0 ura3Δ0*	(3)
FRO-155(T5B)	BY4742 (*MATα his3Δ1 leu2Δ0 lys2Δ0 ura3Δ0 trp5*::I-SceI site-GSHU *lys2*::Alu IR) contains the I-SceI site-GSHU cassette (I-SceI site, I-SceI gene under GAL1 promoter, hygromycin-resistance gene *hyg*, the counterselectable *KlURA3*) and the I-SceI site (HOT site) in *trp5*	(3)
FRO-156(T5B)	*MATα his3Δ1 leu2Δ0 lys2Δ0 ura3Δ0 trp5*::I-SceI site-GSHU *lys2*::Alu IR	(3)
FRO-526(T5)	*MATα his3Δ1 leu2Δ0 lys2Δ0 ura3Δ0* trp5::UK contains the UK cassette (the counterselectable *KlURA3* and *KanMX4* for $G418^R$)	(3)
FRO-527(T5)	*MATα his3Δ1 leu2Δ0 lys2Δ0 ura3Δ0* trp5::UK	(3)
PAT-44	*MATα his3Δ1 leu2Δ0 lys2Δ0 ura3Δ0 trp5*::I-SceI site-GSHU *lys2*::Alu IR *rad52Δ0*	This study
PAT-45	*MATα his3Δ1 leu2Δ0 lys2Δ0 ura3Δ0 trp5*::I-SceI site-GSHU *lys2*::Alu IR *rad52Δ0*	This study
PAT-32(A2B)	*MATα his3Δ1 leu2Δ0 lys2Δ0 ura3Δ0 ade2*::I-SceI site-GSH	This study
PAT-33(A2B)	*MATα his3Δ1 leu2Δ0 lys2Δ0 ura3Δ0 ade2*::I-SceI site-GSH	This study
PAT-42(A2)	*MATα his3Δ1 leu2Δ0 lys2Δ0 ura3Δ0 ade2*::GSH	This study
PAT-43(A2)	*MATα his3Δ1 leu2Δ0 lys2Δ0 ura3Δ0 ade2*::GSH	This study
PAT-34(L2B)	*MATα his3Δ1 lys2Δ0 ura3Δ0 leu2*::I-SceI site-GSH	This study
PAT-35(L2B)	*MATα his3Δ1 lys2Δ0 ura3Δ0 leu2*::I-SceI site-GSH	This study
PAT-36(L2B)	*MATα his3Δ1 lys2Δ0 ura3Δ0 leu2*::I-SceI site-GSH	This study
PAT-37(L2B)	*MATα his3Δ1 lys2Δ0 ura3Δ0 leu2*::I-SceI site-GSH	This study
PAT-38(L2)	*MATα his3Δ1 lys2Δ0 ura3Δ0 leu2*::GSH	This study
PAT-39(L2)	*MATα his3Δ1 lys2Δ0 ura3Δ0 leu2*::GSH	This study
PAT-40(L2)	*MATα his3Δ1 lys2Δ0 ura3Δ0 leu2*::GSH	This study
PAT-41(L2)	*MATα his3Δ1 lys2Δ0 ura3Δ0 leu2*::GSH	This study
55R5-3C	*MAT*a *ura1* ω^- C^R_{321} (*omega*$^-$ chloramphenicol resistant)	(23)
PAT-18(T1B)	*MATα ura1Δ0 trp1*::I-SceI site-GSH	This study
PAT-19(T1B)	*MATα ura1Δ0 trp1*::I-SceI site-GSH	This study
PAT-24(T1)	*MATα ura1Δ0 trp1*::GSH	This study
PAT-25(T1)	*MATα ura1Δ0 trp1*::GSH	This study
PAT-20(L2B)	*MATα ura1Δ0 leu2*::I-SceI site-GSH	This study
PAT-21(L2B)	*MATα ura1Δ0 leu2*::I-SceI site-GSH	This study
BPL-1(L2B)	*MATα ura1Δ0 leu2*::I-SceI site-GSH	This study

BPL-2(L2B)	*MATα ura1Δ0 leu2*::I-SceI site-GSH	This study
PAT-26(L2)	*MATα ura1Δ0 leu2*::GSH	This study
PAT-27(L2)	*MATα ura1Δ0 leu2*::GSH	This study
BPL-4(L2)	*MATα ura1Δ0 leu2*::GSH	This study
BPL-5(L2)	*MATα ura1Δ0 leu2*::GSH	This study
PAT-22(A2B)	*MATα ura1Δ0 ade2*::I-SceI site-GSH	This study
PAT-23(A2B)	*MATα ura1Δ0 ade2*::I-SceI site-GSH	This study
PAT-28(A2)	*MATα ura1Δ0 ade2*::I-SceI site-GSH	This study
PAT-29(A2)	*MATα ura1Δ0 ade2*::I-SceI site-GSH	This study
FRO-767	*leu2*::*HOcs*[1], *mat***a**Δ::*hisG*, *ho*Δ, *hml*Δ::*ADE1*, *hmr*Δ::*ADE1*, *ade1*, *leu2-3,112*, *lys5*, *trp1*::*hisG*, *ura3-52*, *ade3*::*GAL*::*HO*	(24)
HK-225(T5B(HO))	FRO-767 *his3*::*TRP1 leu2*::*ins*[2] *trp5*::*HOcs*	This study
HK-226(T5B(HO))	FRO-767 *his3*::*TRP1 leu2*::*ins trp5*::*HOcs*	This study

Shown are the yeast strains used in this study.

[1]HO cut site.

[2]The *LEU2* open reading frame is replaced by an insert that contains an inverted copy of the *his3* gene, which is expressed by the *GAL1* promoter and is disrupted by an artificial intron, which contains 80 bp of the green fluorescent protein (GFP) gene. Such insertion is not relevant for this study.

Supplementary Table 3 Yeast transformation using A7 aptamer with primers

**Oligo**	**Break on Gal**	
No oligo (*TRP5*)	<0.5	(0-0)
P1-A7-P2.TRP5.40	2,620	(2,250-2,990)
TRP5.40.P1-A7-P2	0.73	(0.28-1.19)

The frequency of Trp^+ colonies per 10^7 viable cells, with mean and 95% confidence intervals (in parentheses), of yeast transformations in the FRO-155 strain from the BY4742 background with the I-SceI gene and with the I-SceI site, using 114-mer oligonucleotides. Two repeats of the no oligo control were done and 6 repeats for the oligonucleotide transformations. Data presented in **Figure 2**.

Supplementary Table 4 Yeast transformation data

A

Oligo	Break on Gal		Break on Glu		No Site on Gal	
No oligo (*TRP5*)	0.54	(0-1.36)	<0.5	(0-0)	<0.5	(0-0)
A7.TRP5.54	62,500	(35,800-89,200)	3.64	(1.00–6.28)	0.16	(0.05-0.27)
C.TRP5.54	8,950	(4,170-13,700)	1.21	(0–2.69)	0.13	(0.03-0.23)
A4.TRP5.54	9,030	(2,120-15,900)	2.09	(1.71-2.47)	<0.4	(0-0)
No oligo (*ADE2*)	<0.5	(0-0)	<0.5	(0-0)	<0.5	(0-0)
A7.ADE2.54	57,900	(41,500-74,300)	15.4	(3.28–27.5)	3.07	(0-7.01)
C.ADE2.54	18,200	(12,800–23,500)	10.9	(0–22.9)	5.03	(0-13.0)
No oligo (*LEU2*)	<0.5	(0-0)	<0.5	(0-0)	<0.5	(0-0)
A7.LEU2.54	522	(333-712)	27.0	(17.1-36.9)	3.61	(1.70-5.52)
C.LEU2.54	253	(163-343)	21.0	(16.0-26.1)	3.74	(1.77-5.71)

B

Oligo	Break on Gal		Break on Glu		No Site on Glu	
No oligo (*TRP1*)	<0.2	(0-0)	<0.2	(0-0)	0.2	(0–0)
A7.TRP1.54	25.8	(18.7-32.8)	10.8	(7.10–14.5)	2.23	(-0.01-4.48)
C.TRP1.54	10.7	(5.77-15.7)	8.75	(4.91–12.6)	4.56	(1.47-7.66)
No oligo (*ADE2*)	<0.2	(0-0)	<0.5	(0-0)	<1	(0-0)
A7.ADE2.54	46.2	(39.4-53.0)	<0.2	(0-0)	<0.4	(0-0)
C.ADE2.54	15.4	(11.9–18.8)	<0.2	(0-0)	<0.4	(0-0)
No oligo (*LEU2*)	<0.2	(0-00)	0.69	(0-3.64)	0.4	(0-0)
A7.LEU2.54	97.1	(73.3-121)	12.8	(4.57-21.1)	8.92	(5.88-12.0)
C.LEU2.54	61.8	(50.4-73.2)	12.5	(4.96-20.0)	7.90	(5.38-10.4)

(**A**) The frequency of transformant colonies per 10^7 viable cells, with mean and 95% confidence intervals (in parentheses), of yeast transformations in strains from the BY4742 background. For the strains with I-SceI expression and the I-SceI site (Break on Gal), 12 to 18 repeats of each transformation were performed (with the exception of the negative controls, for the *trp5* locus no oligo control there were 6 repeats, and for the *ade2* locus and *leu2* locus no oligo controls there were 3 repeats). Data presented in **Figure 3A**. For the strains without the I-SceI site present (and in the case of the *trp5* locus, no I-SceI gene) and grown on galactose media (No Site on Gal), 8 to 12 repeats were performed of each transformation, except for the no oligo controls and the A4.TRP5.54 oligonucleotide, which were repeated 3 times. Data presented in **Figure 3B**. For the strains with the I-SceI gene and site, but grown on glucose media such that no I-*Sce*I protein would be expressed (Break on Glu), 4 repeats of the A7.TRP5.54 and C.TRP5.54 oligonucleotides were performed and 7 repeats of the A4.TRP5.54 oligonucleotide was performed for the *trp5* locus. For the *ade2* and *leu2* loci, 12 repeats of the A7 aptamer containing oligonucleotide (the A4 aptamer was not tested at these loci) and the non-binding control containing oligonucleotide were used. Two repeats of the no oligo control were performed for the *trp5* and *ade2* loci, while 3 repeats of the no oligo control were performed for the *leu2* locus. Data presented in **Figure 3C**.

(**B**) The frequency of transformant colonies per 10^7 viable cells, with mean and 95% confidence intervals (in parentheses), of yeast transformations in strains from the 55R5-3C background with or without the I-SceI gene and with or without the I-*Sce*I site. For the strains with I-SceI expression and the I-SceI site (Break on Gal), 23 to 29 repeats of each transformation were performed (with the exception of the negative controls, for the *trp1* locus no oligo control there were 7 repeats, for the *ade2* locus there were 6 repeats, and for the *leu2* locus no oligo controls there were 5 repeats). Data presented in **Figure 4A**. For the strains without the I-SceI site present and grown on glucose media (No Site on Glu), 25 repeats were performed of each transformation for the *trp1* locus, except for the no oligo control which was repeated 5 times. For the *ade2* locus, 3 repeats of the transformations were done and one repeat of the no oligo control. For the *leu2* locus, 9 repeats of each of the transformations were done and 3 repeats of the no oligo control. Data presented in **Figure 4B**. For the strains with the I-SceI gene and site, but grown on glucose media such that no I-SceI protein would be expressed (Break on Glu), 25 repeats of the A7.TRP1.54 and C.TRP1.54 oligo were performed for the *trp1* locus, 6 repeats of A7.ADE2.54 and C.ADE2.54 were performed for the *ade2* locus, and 9 repeats for the *leu2* locus. Two repeats of the no oligo control were performed for the *ade2* locus, while 3 repeats of the no oligo control was performed for the *leu2* locus, and 5 repeats of the no oligo control was done for the *trp1* locus. Data presented in **Figure 4C**.

Supplementary Table 5 Yeast transformations with shorter oligos

Oligo	Break on Gal		Oligo	Break on Gal	
No oligo (*TRP5*)	<0.5	(0-0)	No oligo (*ADE2*)	<0.4	(0-0)
A7.TRP5.40	202	(155-248)	A7.ADE2.40	404	(311-496)
C.TRP5.40	13.9	(8.13-19.4)	C.ADE2.40	464	(316–612)
No oligo (*LEU2*)	<0.3	(0-0)	NT.TRP5.40	<0.3	(0-0)
A7.LEU2.40	8.63	(5.84-11.4)	NT.ADE2.40	6.28	(2.18-10.4)
C.LEU2.40	12.89	(8.06-17.7)	NT.LEU2.40	0.66	(0-1.37)

The frequency of transformant colonies per 10^7 viable cells, with mean and 95% confidence intervals (in parentheses), of yeast transformations with strains from the BY4742 background containing both the I-SceI gene and the I-SceI site. The purpose of these experiments was to test oligonucleotides that contained the full length A7 aptamer but with a shorter homology region. For the no oligo controls, there were two repeats for the *trp5* locus, three repeats for the *ade2* locus, and four repeats for the *leu2* locus. The transformations involving the oligonucleotides with 40 bases of homology were repeated 10 to 16 times, except for those oligonucleotides that contained only homology (NT.TRP5.54, NT.ADE2.54, and NT.LEU2.54) to the target locus, which were repeated 4 to 8 times. Data presented in **Figure 3D**.

Supplementary Table 6 PAGE purified oligonucleotides

Purified Oligo	Break on Gal	
No oligo (*TRP5*)	1.02	(0-2.21)
A7.TRP5.54-P	1,340	(594-2,090)
C.TRP5.54-P	51.0	(16.5-85.5)
A7.TRP5.40-P	122	(72.7-171)
C.TRP5.40-P	3.78	(2.93-4.62)

Polyacrylamide gel electrophoresis (PAGE) purified oligonucelotides were ordered and tested at the *trp5* locus in the FRO-155 strain that contains both the I-SceI gene and the I-SceI site. The frequency of Trp^+ colonies per 10^7 viable cells, with mean and 95% confidence intervals (in parentheses), of the yeast transformations are shown. The no oligo control was repeated 4 times and the oligonucleotide transformations were repeated 5 to 9 times. Data presented in **Figure 3E**.

Supplementary Table 7 *trans* assay

Oligo	Break on Gal	
No oligo (*LEU2*)	<0.5	(0-0)
A7.TRP5.40	<0.5	(0-0)
C.TRP5.40	<0.5	(0-0)
C.LEU2.54	193	(172-221)
C.LEU2.40	8.89	(5.71-12.1)
A7.TRP5.40 + C.LEU2.54	178	(103-252)
A7.TRP5.40 + C.LEU2.40	11.9	(7.07-16.6)
C.TRP5.40 + C.LEU2.54	186.9	(127-246)
C.TRP5.40 + C.LEU2.40	12.5	(6.52-18.4)

Oligonucleotides tested at the *leu2* locus in the PAT-34 and PAT-35 strains that contain both the I-SceI gene and the I-SceI site. The frequency of Leu^+ colonies per 10^7 viable cells, with mean and 95% confidence intervals (in parentheses), of the yeast transformations are shown. The controls (No oligo and the individual oligonucleotides) were repeated 2 times and the transformations with two oligonucleotides were repeated 8 times. Data presented in **Figure 3F**.

Supplementary Table 8 Specificity of the I-SceI aptamer

Oligo	Break on 2.0% Gal		Break on 0.2% Gal	Break on Glu	
	I-SceI	HO	HO	I-SceI	HO
No oligo (*TRP5*)	<0.3 (0-0)	<0.3 (0-0)	<0.3 (0-0)	<0.3 (0-0)	<0.3 (0-0)
A7.TRP5.40	72.4 (44.4-100)	138,487 (117,690-159,283)	15,112 (12,525-17,701)	0.67 (0.23-1.1)	3.6 (2.3-5.0)
C.TRP5.40	7.9 (2.5-13.3)	69,042 (42,236-95,849)	9558 (7454-11,662)	0.42 (0-0.93)	1.8 (0-4.0)

The frequency of Trp^{+} colonies per 10^{7} viable cells, with mean and 95% confidence intervals (in parentheses). All transformations were repeated 4 times. Data presented in **Figure 3G**.

Supplementary Table 9 DsRed2 transfection data

Transfected DNA	Flow Cytometry		Hand Counts	
No DNA	0.25	(0-0.84)	<0.4	(0-0)
pSce	0.75	(0-1.55)	<0.4	(0-0)
pLDSLm	0.50	(0-1.42)	<0.4	(0-0)
pSce + pLDSLm	1.25	(0.45-2.05)	<0.4	(0-0)
A7.Red.54	<0.3	(0-0)	<0.4	(0-0)
C.Red.54	0.75	(0-3.14)	<0.4	(0-0)
A7.Red.40	0.75	(0-2.27)	<0.4	(0-0)
C.Red.40	1.75	(0-5.28)	<0.4	(0-0)
A7.Red.30	<0.3	(0-0)	<0.4	(0-0)
C.Red.30	0.5	(0-1.42)	<0.4	(0-0)
**A7.Red.54**	49.3	(34.8-63.8)	1,040	(689-1,400)
**C.Red.54**	30.0	(21.7-38.2)	554	(355-753)
**A7.Red.40**	39.7	(24.5-54.9)	411	(272-550)
**C.Red.40**	7.39	(4.30-10.5)	66.2	(28.7-104)
**A7.Red.30**	14.3	(6.60-22.0)	199	(77.8-320)
**C.Red.30**	3.92	(1.62-6.20)	12.4	(3.74-21.1)
**NT.Red.40**	4	(2.04-5.96)	6.78	(1.04-12.5)
**NT.Red.30**	0.4	(0-1.08)	N/A	

The frequency of RFP^+ cells per 100,000 cells (flow cytometry) or RFP^+ cells per well given 150,000 cells seeded (hand counts), with mean and 95% confidence intervals (in parentheses), of HEK-293 transfections. The oligonucleotide transfections with both the I-SceI expression vector and the target plasmid are bolded. Cells were transfected with an I-SceI expression vector (pSce) and a target plasmid that contains the DsRed2 gene disrupted by two STOP codons and an I-SceI site. No DNA control, pSce only, pLDSLm only, and the oligos only negative controls were repeated 4 to 8 times. The 54 base homology containing oligonucleotide transfections using both plasmids

(A7.Red.54 and C.Red.54) were repeated thirty-eight times, the oligonucleotides with 40 bases of homology (A7.Red.40 and C.Red.40) and the oligonucleotides with 30 bases of homology (A7.Red.30 and C.Red.30) were repeated 13 or 14 times, and the oligonucleotides that contained only homology with no 5' aptamer or non-binding control sequence (NT.Red.40 and NT.Red.30) were repeated 5 times. The flow cytometer was thought to be overreporting the number of RFP^+ cells and hence underreporting the difference between the oligonucleotides with 30 bases of homology, as even the negative control samples contained some RFP^+ cells according to the flow cytometer. Data presented in **Figure 5A**.

In order to further validate the overreporting of RFP^+ cells by the flow cytometer, hand counts of the transfections were done. After seeding 150,000 cells, after 5 to 8 days the numbers of RFP^+ cells were counted in each well. For the negative controls which were repeated 3 times each, no RFP^+ cells were seen. The oligonucleotides with both the I-SceI expression vector and the target plasmid (shown bolded above) were repeated 12 to 14 times. Data presented in **Figure 5B**.

Supplementary Table 10 DsRed2 digested pLDSLm transfection data

Transfected DNA	Flow Cytometry	
No DNA	2	(2-2)
Dig. pLDSLm	<0.5	(0-0)
A7.Red.54	1	(1-1)
C.Red.54	3	(3-3)
A7.Red.40	1	(1-1)
C.Red.40	4	(4-4)
A7.Red.30	1	(1-1)
C.Red.30	1	(1-1)
**A7.Red.54**	737	(653-821)
**C.Red.54**	416	(265-567)
**A7.Red.40**	235	(124-346)
**C.Red.40**	144	(96.0-192)
**A7.Red.30**	143	(101-185)
**C.Red.30**	137	(86.3-188)

The frequency of RFP^+ cells per 100,000 cells (flow cytometry), with mean and 95% confidence intervals (in parentheses), of HEK-293 transfections. The oligonucleotide transfections with the target plasmid are bolded. Cells were transfected with a target plasmid that contained the DsRed2 gene disrupted by two STOP codons and an I-SceI site that was digested by I-SceI *in vitro* prior to transfection. No DNA control, I-SceI digested pLDSLm only, and the oligonucleotide only negative controls were repeated 1 to 2 times. The oligonucleotide transfections with the digested pLDSLm vector (bolded) were repeated 5 times. Data presented in **Figure 5C**.

Supplementary Table 11 Yeast transformation in a *rad52*Δ background

Oligo	Break on Gal
No oligo (*TRP5*)	2.14 (1.27-3.01)
A7.TRP5.54	3.06 (2.47-3.6)
C.TRP5.54	2.57 (1.73-3.42)

The frequency of Trp^+ colonies per 10^7 viable cells, with mean and 95% confidence intervals (in parentheses), of yeast transformations in the FRO-155 strain from the BY4742 background with the I-SceI gene and with the I-SceI site, except that the *RAD52* gene has been deleted. 8 repeats of the no oligo control were done and 12 repeats for the A7.TRP5.54 and C.TRP5.54 oligonucleotides. Data presented in **Supplementary Figure 5**.

Supplementary Figure Legends

Supplementary Figure 1 DNA library run without I-SceI protein and the bulk affinity assay with I-SceI CE electropherograms showing key steps of the aptamer selection, the X axis shows the time each sample took to reach the detector and the Y axis shows the amount of DNA measured by the relative fluorescence units (RFU). A) The random single-stranded DNA library (1 μM) was run in the absence of I-SceI. The free DNA begins to appear at approximately 10 minutes. The relative fluorescence units (RFU) measure the amount of the FAM-labeled DNA. There are no complexes prior to 10 minutes, as shown in a zoomed-in picture displayed in the box to the right. B) An initial bulk affinity assay was performed with 1.5 μM I-SceI and 100 nM DNA in order to view any I-SceI-DNA complexes. I-SceI-DNA complexes were observed prior to the free DNA peak at ~10 minutes. A zoomed-in picture of the complexes is displayed in the box to the right.

Supplementary Figure 2 Non-SELEX rounds 1, 2, and 3 and the I-SceI aptamer A7 A series of CE runs showing the first 3 rounds of selection, as well as the CE run of the A7 aptamer with I-SceI. The X axis shows the time each sample took to reach the detector and the Y axis shows the amount of DNA measured by the relative fluorescence units (RFU). A) In the first round of selection, I-SceI-DNA complex peaks could be detected prior to the free DNA peak at ~10 minutes. A zoomed-in picture of the complexes is displayed in the box to the right. The second round of selection was done using the complexes collected in the first round of selection. B) For round 2, the amount of total DNA drastically decreased while the ratio of DNA in complex with I-SceI compared to the total DNA increased. C) In the third round of selection the amount of total DNA was very low, and no complex could be detected. D) One of the selected aptamers, A7, run with I-SceI. 50nM PAGE purified, FAM-labeled A7 was run with 1μM of dialyzed I-SceI. The I-SceI-DNA complex formed with I-SceI is boxed and the unbound DNA is boxed with dotted lines. Due to the single-stranded nature of the DNA sequence, there is a broad peak for the unbound DNA not complexed with I-SceI due to self-hybridization and concatenation between the oligonucleotides.

Supplementary Figure 3 Weak, moderate, and strong binding aptamers to I-SceI An example of the several different types of I-SceI candidate aptamers screened is shown. The X axis represents the time each sample took to reach the detector and the Y axis shows the amount of DNA measured by the relative fluorescence units (RFU). Weak binders (boxed in blue) showed very small complex peaks or no complex peaks when ran in CE-LIF with I-SceI, whereas moderate binders (boxed in yellow) showed small to medium-sized I-SceI-DNA complex peaks. Sequences that bound strongly to I-SceI (boxed in red) showed large I-SceI-DNA complex peaks. Complex peaks are within the dotted boxes. There is a large peak in each electropherogram that is not an I-SceI-DNA complex, but is due to the presence of the leftover FAM-labeled P1 primer from the asymmetric PCR.

Supplementary Figure 4 EMSA screen of I-SceI aptamers reveals A7 and A4 as binders to I-SceI A polyacrylamide gel showing several aptamers, including both the A4 and A7 aptamer that were further characterized for binding to I-SceI. A sequence containing the primers from the random DNA library but with an internal sequence not selected to bind to I-

SceI was used as a negative control (P1-r-P2). Some DNA was unable to enter into the gel and remained as DNA aggregates in the top of the wells.

Supplementary Figure 5 Aptamer-guided gene targeting is Rad52 dependent Frequency of gene correction by the aptamer-containing oligonucleotide (A7.TRP5.54 shown in light gray) or the non-binding control oligonucleotide (C.TRP5.54 shown in dark gray) (X axis) was measured by the number of Trp^+ transformants per 10^7 viable cells (Y axis) in a *rad52*Δ FRO-155 (T5B) background strain. Bars correspond to the mean value and error bars represent 95% confidence intervals. Asterisks denote statistical significant difference between the aptamer-containing oligonucleotide and the no oligonucleotide negative control (* for $p<0.05$, ** for $p<0.01$, *** for $p <0.001$, and **** for $p<0.0001$). For additional information see **Supplementary Table 11**.

Supplementary Figure 1

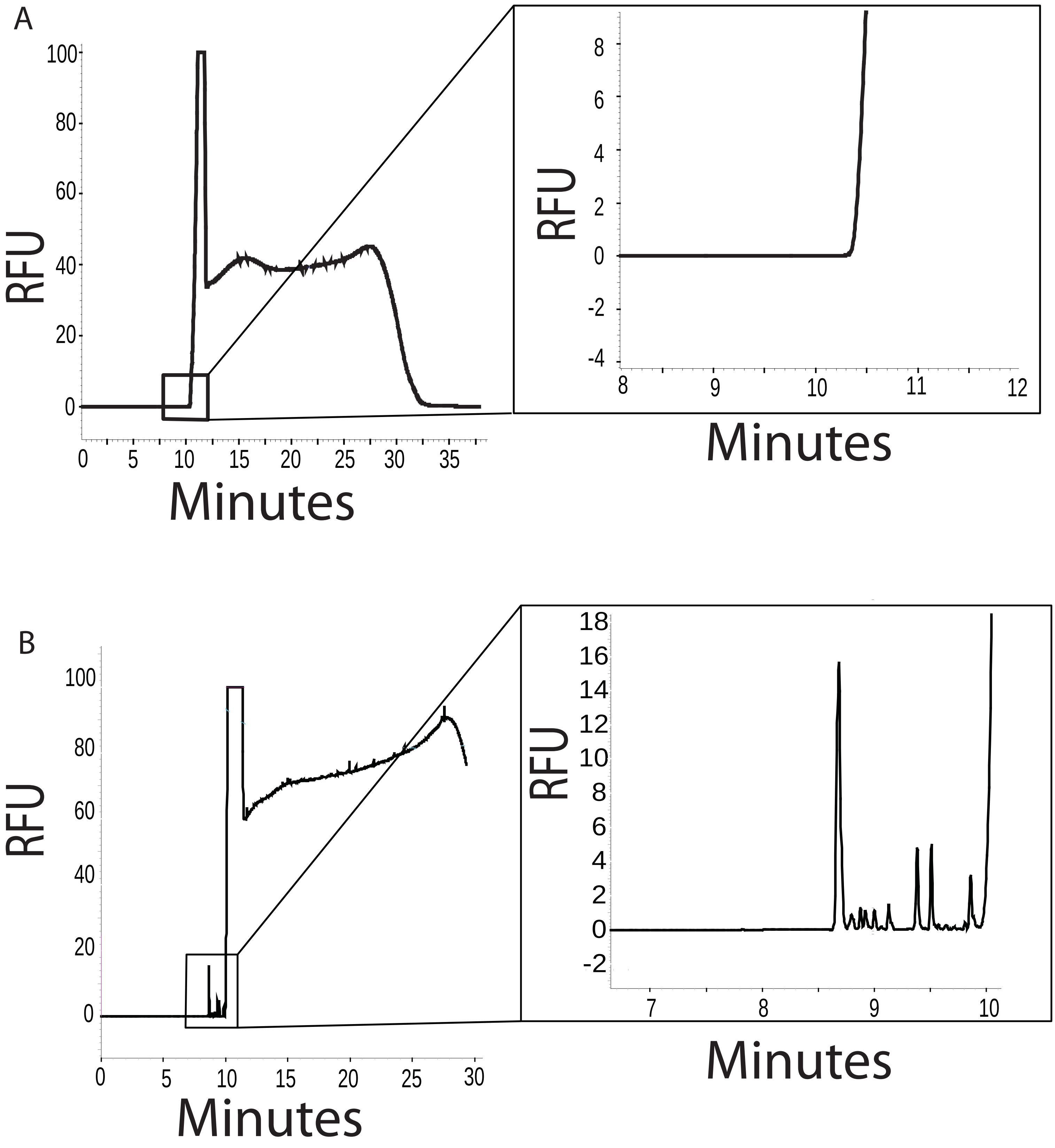

Supplementary Figure 2

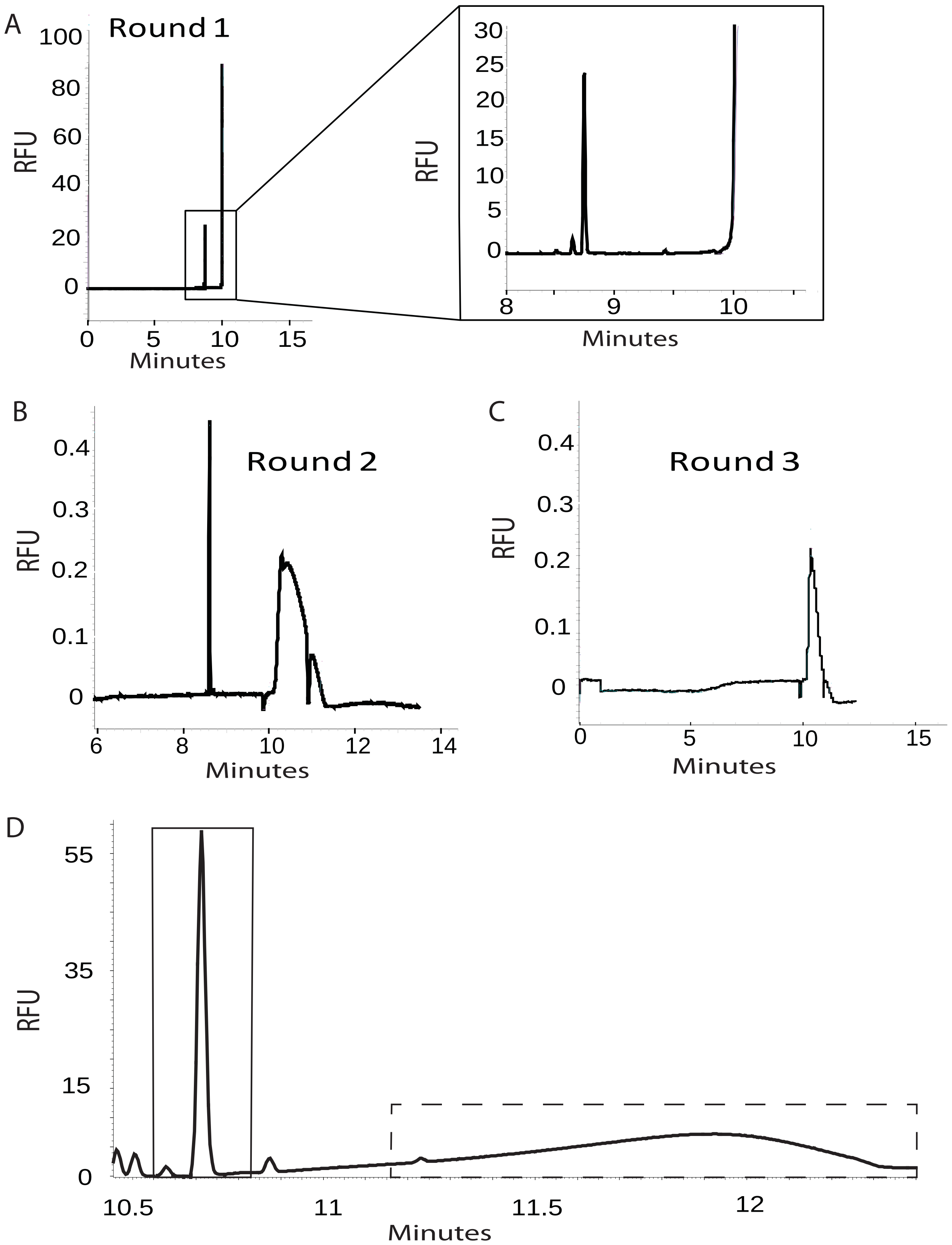

Supplementary Figure 3

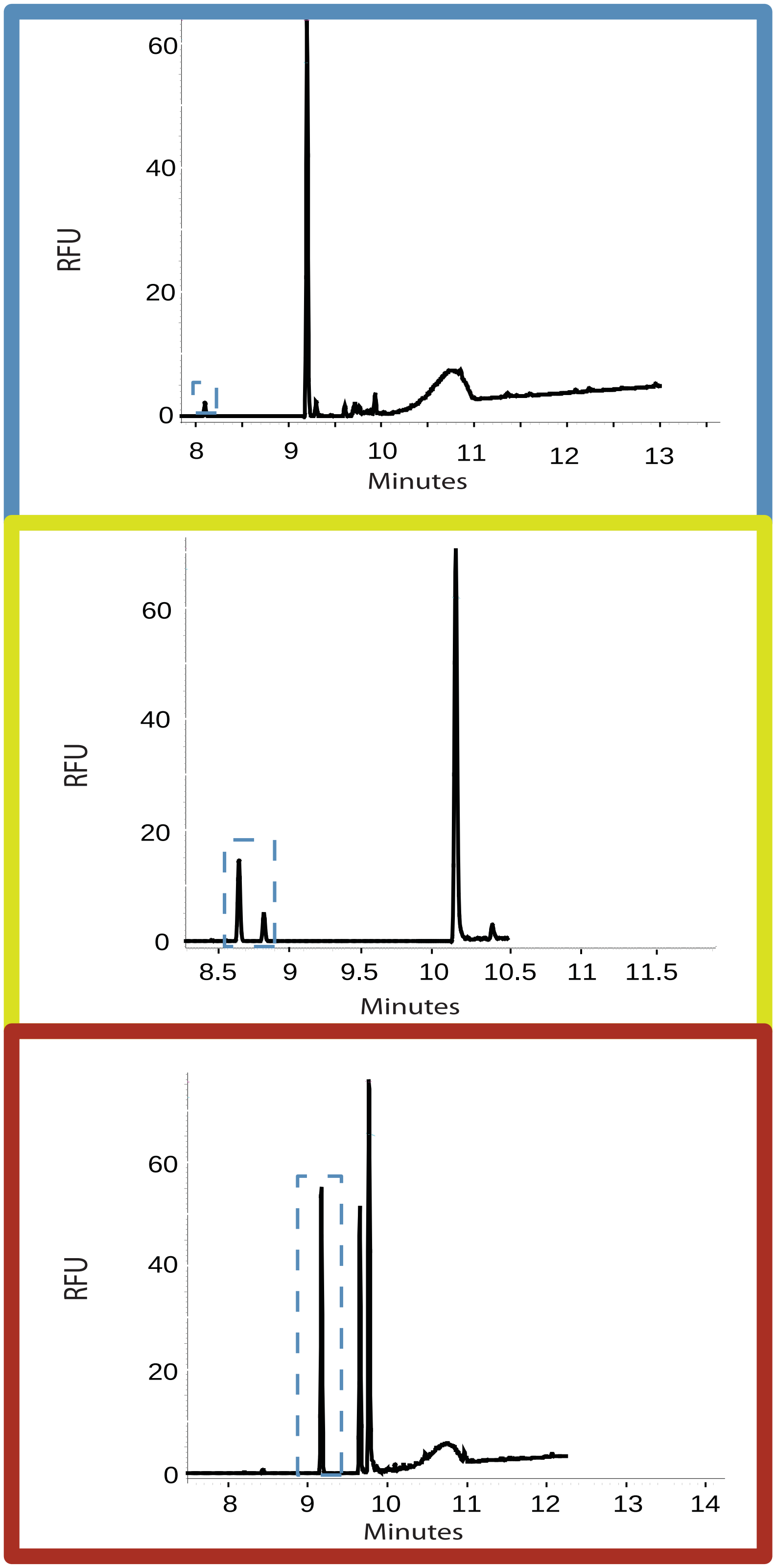

Supplementary Figure 4

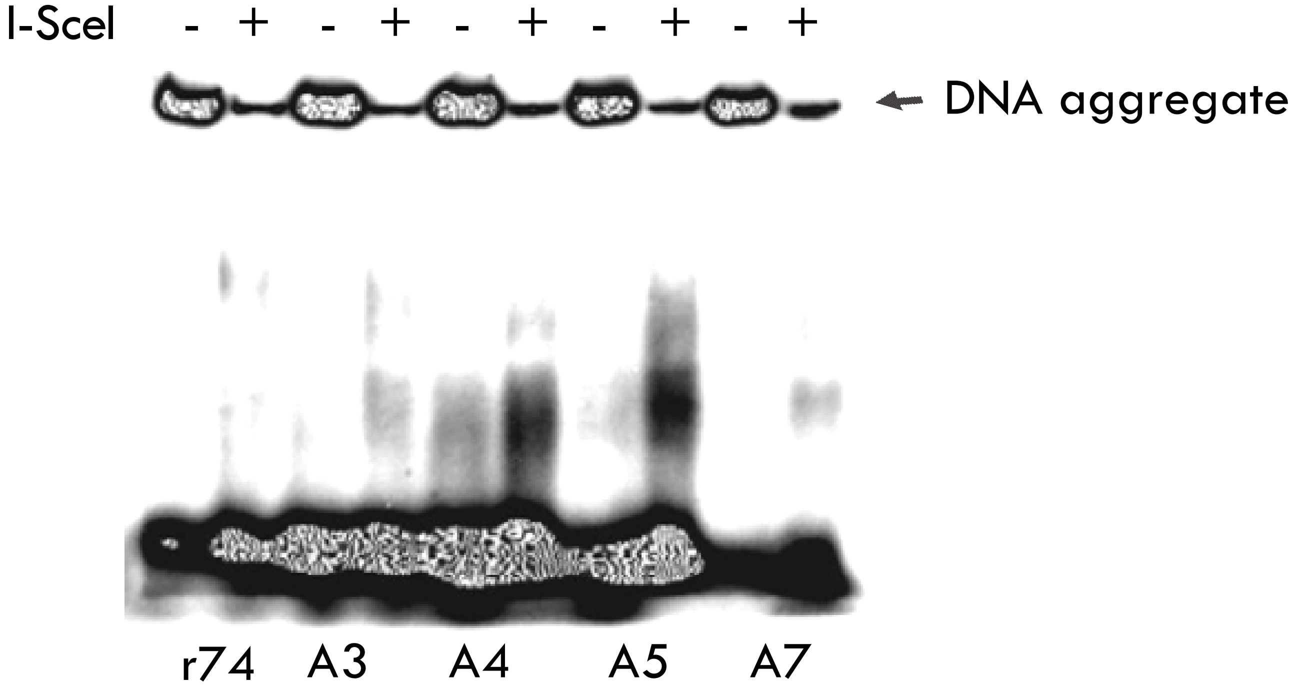

Supplementary Figure 5

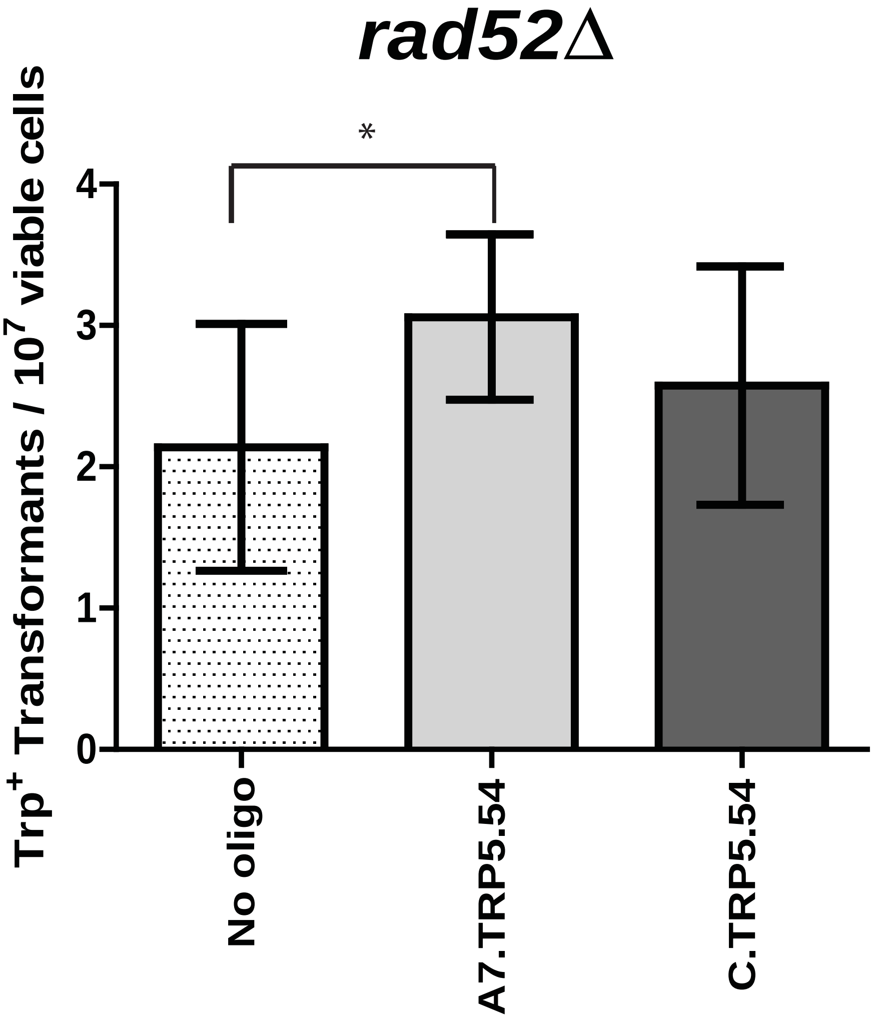

Supplement: Supplementary Data [file supp_gku101_nar-02125-met-g-2013-File009.pdf]
